# The MdVQ22‐MdWRKY33d complex regulates *MdPER55* and *MdDREB1D* expression to modulate apple salt tolerance by reactive oxygen species scavenging

**DOI:** 10.1111/tpj.71191

**Published:** 2026-10-23

**Authors:** Hongmin Hou, Shuxin Li, Qi Zhang, Guo Chen, Wengang Wu, Xueli Liu, Shenghui Jiang, Jihua Xu, Xiao Liang, Dingchang Meng, Abdulqahhor Toshpulatov Khusniddinugli, Farrukh Matkarimov Ilkhomovich, Yugang Zhang

**Affiliations:** ^1^ College of Horticulture Qingdao Agricultural University Qingdao China; ^2^ Engineering Laboratory of Genetic Improvement of Horticultural Crops of Shandong Province Qingdao Agricultural University Qingdao China; ^3^ National Center of Technology Innovation for Comprehensive Utilization of Saline‐Alkali Land Dongying 257347 China; ^4^ Academy of Dongying Efficient Agricultural Technology and Industry on Saline and Alkaline Land in Collaboration with Qingdao Agricultural University Dongying China; ^5^ College of Life Sciences Qingdao Agricultural University Qingdao China; ^6^ Institute of Genetics and Plant Experimental Biology Academy of Sciences of Uzbekistan Yukori‐yuz, Kibray Uzbekistan

**Keywords:** apple, salt stress, WRKY transcription factor, VQ protein, ROS scavenging

## Abstract

Apple is a globally significant fruit tree with considerable economic value. However, it is severely affected by soil salinization, which leads to a reduction in both yield and quality. Since salt tolerance in grafted apple trees largely depends on the rootstock, this study compared transcriptomic responses between salt‐tolerant (R5‐5) and salt‐sensitive (M9T337) rootstocks under salt stress. A key salt‐responsive gene, *MdWRKY33d*, was identified. Overexpressing *MdWRKY33d* in apple improved salt tolerance, while silencing it had the opposite effect. Additionally, under salt stress, compared with the control, chlorophyll fluorescence values, superoxide dismutase (SOD), peroxidase (POD), and catalase (CAT) levels were elevated in *MdWRKY33d* transgenic apple and poplar materials. Functional analyses demonstrated that *MdWRKY33d* directly activates the expression of *Peroxidase55* (*MdPER55*) and *Dehydration‐Responsive Element‐Binding protein1D* (*MdDREB1D*), thereby enhancing reactive oxygen species (ROS) scavenging and salt tolerance in transgenic plants. Yeast two‐hybrid screening identified MdVQ22 as an interacting partner of MdWRKY33d, which was confirmed by pull‐down, bimolecular fluorescence complementation (BiFC), and luciferase complementation imaging (LCI) assays. *MdVQ22* negatively regulated apple salt tolerance by suppressing MdWRKY33d‐mediated transcriptional activation of *MdPER55* and *MdDREB1D*. Collectively, this study explores a putative regulatory module MdVQ22‐MdWRKY33d‐*MdPER55*/*MdDREB1D* to coordinate the responses to apple salt stress and provides potential targets for engineering salt‐tolerant varieties.

## Significance Statement

Apple (*Malus domestica*) is one of the most extensively cultivated fruit trees worldwide, playing a crucial role in agricultural economies. However, soil salinization remains a significant challenge that severely restricts apple production, especially in regions affected by secondary salinization. This study reveals a regulatory module consisting of MdVQ22‐MdWRKY33d‐*MdPER55*/*MdDREB1D* that modulates apple salt stress responses, providing theoretical insights for breeding salt‐tolerant apple rootstocks.

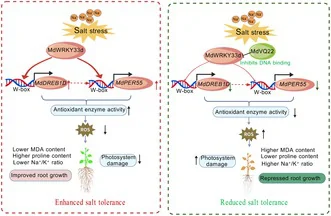

## INTRODUCTION

Apple (*Malus domestica* Borkh.) is one of the most economically valuable fruit trees globally. However, its growth and development are adversely impacted by various abiotic stresses. Salt stress, as the most widely distributed abiotic stressor worldwide, not only severely constrains the geographical distribution of apple trees but also significantly reduces both apple yield and fruit quality (Van Zelm et al., 2020). According to the Food and Agriculture Organization of the United Nations (FAO, 2024), the global saline soil area has approximately reached 1.381 billion hectares, accounting for 10.7% of the world's total land area. Therefore, elucidating the molecular mechanisms of apple salt tolerance and developing salt‐resistant cultivars/rootstocks are crucial for advancing the apple industry.

Salt stress induces the excessive production of reactive oxygen species (ROS), including hydrogen peroxide (H_2_O_2_), superoxide anion (O_2_^−.^), and hydroxyl radical (HO·) (Bose et al., 2013). The overaccumulation of intracellular ROS causes oxidative damage to DNA, RNA, proteins, and biomembranes, thereby disrupting cellular homeostasis (Miller et al., 2009). To counteract such damage, plants have evolved a multi‐layered antioxidant defense system comprising enzymatic and non‐enzymatic components. Key antioxidant enzymes comprise superoxide dismutase (SOD), peroxidase (POD), and catalase (CAT), whereas nonenzymatic antioxidants include ascorbic acid (AsA), glutathione (GSH), and flavonoids (Van Zelm et al., 2020). Peroxidases (PRXs), as core enzymes in ROS scavenging, play crucial roles in plant salt stress resistance. Studies have shown that PRXs significantly enhance plant tolerance to salt stress by directly eliminating ROS or participating in redox signaling transduction (Jiao et al., 2024; Su et al., 2020; Van Zelm et al., 2020; Zhai et al., 2024; Zhang, Wang, et al., 2022). Dehydration‐responsive element binding (DREB) proteins, which belong to the AP2/ERF superfamily, play a crucial role in plant stress responses (Agarwal et al., 2006; Sakuma et al., 2002; Zhang & Xia, 2023). They specifically bind to DRE cis‐acting elements in the promoters of various downstream stress‐response genes, thereby regulating their expression and modulating abiotic stress tolerance. In Arabidopsis, DREB family members are categorized into six groups, A‐1 to A‐6, with distinct functions (Nakano et al., 2006; Sakuma et al., 2002). Notably, earlier studies showed that the DREB1/CBF genes in the A‐1 group are involved in responses to salt stress. For example, the *AtDREB1F* (DDF1) gene, which is located on a branch closely related to the DREB1/CBF family, responds to high salinity (Magome et al., 2004). Furthermore, transgenic plants overexpressing *AtDDF1* have exhibited increased tolerance to high‐salinity stress. Moving beyond Arabidopsis, in rice, overexpression of *OsDREB1D* enhances the high‐salt tolerance of transgenic Arabidopsis (Zhang et al., 2009). Additionally, *OsDREB1B* enhances salinity tolerance in rice seedlings by elevating the contents of soluble sugars and proline (Liu et al., 2025). In soybeans, the GmDREB1B protein can bind to the DRE/CRT (C‐repeat, with the sequence GCCGAC) in the promoter region of the *GmDof41* gene, thereby enhancing the salt and drought tolerance of soybeans (Kidokoro et al., 2015; Wei et al., 2023). In sweet cherry, the *PaDREB1*‐overexpressing transgenic plant exhibited greater salt tolerance than the wild‐type (Kitashiba et al., 2004). In apple, *MbDREB1* increases plant tolerance to salt stress via both ABA‐dependent and ABA‐independent pathways (Yang et al., 2010). Similarly, we also found that *MdDREB1D* showed a strong response to salt stress in the apple rootstock salt‐stress transcriptome data and positively regulated salt tolerance in apple by improving ROS scavenging.

In this study, WGCNA analysis of RNA‐Seq data from salt‐tolerant (R5‐5) and salt‐sensitive (M9T337) apple rootstocks under salt stress revealed a significant positive correlation between the MEblue module and the activities of superoxide dismutase (SOD), peroxidase (POD), and catalase (CAT). Within the MEblue module, *MdWRKY33d* was identified as a salt‐responsive regulator whose overexpression enhances apple salt tolerance by upregulating antioxidant enzymes to scavenge ROS. WRKY transcription factors constitute one of the largest families of transcriptional regulators, playing important roles in plant growth and development, as well as in responses to biotic and abiotic stresses (Wang, Li, et al., 2025; Yang et al., 2025). Currently, WRKY transcription factors associated with salt stress have also been isolated and characterized in various plant species (Krishnamurthy et al., 2020; Lan et al., 2025; Lu et al., 2023; Shang et al., 2024; Wang, Wei, et al., 2025; Yu et al., 2023; Zhou et al., 2024). In apple, although some WRKY transcription factors have been reported to participate in the salt stress response (Han et al., 2018; Li et al., 2025; Wang, Shan, et al., 2025; Zhao et al., 2025), the potential salt‐tolerant functions and regulatory mechanisms of numerous WRKY genes remain to be elucidated.

(Valine‐glutamine) VQ proteins are a class of transcriptional regulatory cofactors that contain VQ domains, which are characterized by the presence of 10 highly conserved amino acid residues with the sequence pattern FxxhVQxhTG (Cheng et al., 2012; Jing & Lin, 2015). Currently, research on the interaction between VQ proteins and WRKY transcription factors is extensive. They collaboratively participate in the regulation of specific biological processes and biotic stress responses (Dong et al., 2024; Duan et al., 2025; Hu et al., 2013; Huang et al., 2024; Lin et al., 2025; Tian et al., 2023; Yang et al., 2024). Additionally, some studies have demonstrated that they can work in concert to participate in the plant's response to abiotic stress. For example, AtVQ9 antagonizes AtWRKY8 to modulate Arabidopsis salinity tolerance by attenuating AtWRKY8 binding affinity for the *AtRD29A* promoter. (Hu et al., 2013). Similarly, the sweet potato IbVQ4‐IbWRKY2 complex enhances salt tolerance in heterologous Arabidopsis by activating the ABA pathway (Zhu et al., 2020). In tomato, SlVQ16 and SlVQ21 competitively bind SlWRKY57 to antagonize its repression of *SlRD29B* and *SlSOS1*, thereby finely tuning salt tolerance (Ma et al., 2023). Beyond salinity, the BcVQ11A‐BcWRKY23‐BcWRKY25 module regulates thermotolerance in non‐heading Chinese cabbage via phenylalanine ammonia‐lyase activity (Gao et al., 2024). However, molecular‐level research on how VQ‐WRKY complexes regulate abiotic stress resistance in apple remains limited. To date, only the MdWRKY75‐MdVQ10 module has been shown to accelerate wound‐induced leaf senescence by transactivating *MdSAG12* and *MdSAG18* (Zhang et al., 2023).

In this study, the salt‐responsive regulator *MdWRKY33d* improves apple salt tolerance via activation of antioxidant enzymes and scavenging of ROS. Further analysis revealed that *MdWRKY33d* directly promotes the expression of *MdPeroxidase55* (*MdPER55*) and *MdDREB1D*. Additionally, the MdVQ22 protein, which physically associates with MdWRKY33d, negatively modulates apple salt tolerance by suppressing MdWRKY33d‐mediated transcriptional activation of *MdPER55* and *MdDREB1D*. Overall, this research elucidated the molecular mechanisms of the MdVQ22‐MdWRKY33d‐*MdPER55*/*MdDREB1D* module in coordinating apple salt stress responses, providing genetic resources and theoretical insights for breeding salt‐tolerant apple rootstocks.

## RESULTS

### Phenotypic and physiological changes in two apple rootstock varieties under salt stress

Rootstocks are vital for apple trees, especially in dealing with salt stress. We compared salt‐tolerant apple rootstock line R5‐5 with the salt‐sensitive apple rootstock line M9T337 (both healthy and similarly grown) under normal and 150 mM NaCl salt stress for 15, 20, and 25 days. While both rootstocks grew well under normal cultivation conditions, salt stress inhibited their growth to varying degrees. After 20 days under salt stress, M9T337's old leaves yellowed and curled, exhibiting symptoms of salt injury, while R5‐5 remained unaffected (Figure S1A). By the 25th day, M9T337 leaves had severely deteriorated, whereas R5‐5 only exhibited minor yellowing in young leaves, demonstrating stronger salt tolerance (Figure S1A). Consistent with phenotypic observations, following salt stress, R5‐5 exhibited significantly higher net photosynthetic rate (Pn), stomatal conductance (g_s_), and transpiration rate (E) compared to M9T337, along with a lower intercellular carbon dioxide concentration (Ci). This suggests that R5‐5 can maintain relatively high photosynthetic efficiency under salt stress (Figure S1B–E). In addition, under salt stress, the M9T337 roots suffered a more severe inhibition, with some roots displaying twisted and deformed morphological traits (Figure S2A). Conversely, R5‐5 exhibited greater root length, surface area, volume, and vitality compared to M9T337, while showing lower relative electrical conductivity and malondialdehyde (MDA) content (Figure S2B–G). Similarly, R5‐5 leaves and roots accumulated significantly less Na^+^ and more K^+^ than M9T337, leading to a lower Na^+^/K^+^ ratio (Figure S1F,G and Figure S2H–J). In summary, following salt stress, the R5‐5 rootstock suffered relatively less damage, demonstrating stronger salt tolerance and adaptability compared to the M9T337.

### *MdWRKY33d* is a positive regulator in response to salt stress in apple

To investigate the key regulatory genes involved in the response to salt treatments, we conducted transcriptomic sequencing on the roots of R5‐5 (salt‐tolerant) and M9T337 (salt‐sensitive), which were subjected to 150 mM salt treatments for 0 and 48 h. Based on the RNA‐Seq data, weighted gene co‐expression network analysis (WGCNA) was used to analyze the correlation between differentially expressed genes (DEGs) and physiological indices, such as superoxide dismutase (SOD), peroxidase (POD), catalase (CAT), malondialdehyde (MDA), and proline (Pro), in the two varieties under salt stress (Figure 1A–C). WGCNA clustered the DEGs into nine modules (Figure 1A,B). The correlation analysis between modules and physiological indices showed that the MEblue module was significantly positively correlated with SOD, POD, and CAT activity, and Pro content (Figure 1C). The DEGs in the module were enriched in pathways including MAPK signaling pathway‐plant, phenylpropanoid biosynthesis, Plant−pathogen interaction, and flavonoid biosynthesis (Figure 1D). Furthermore, we found multiple WRKY transcription factors in these three pathways. This indicates that the WRKY genes within this module play a crucial role in apple response to salt stress. Further analysis showed that MD04G1167700 and MD12G1181000 were significantly upregulated in R5‐5 compared to M9T337 under salt stress (Figure 1E). Phylogenetic tree analysis revealed that MD04G1167700 and MD12G1181000 are homologous genes, and both exhibit the closest evolutionary relationship with Arabidopsis *AtWRKY33* (Figure S3A). Sequence alignment confirmed that they both possess two conserved WRKY domains and C2H2 zinc finger domains, belonging to the WRKY‐I subfamily (Figure S3A,B). We chose MD04G1167700 with a higher FPKM value, naming it *MdWRKY33d* to carry out the following experiments (Figure 1E). Consistent with the transcriptome data, qRT‐PCR results also demonstrated that *MdWRKY33d* in R5‐5 exhibited significantly higher expression levels compared to M9T337 during the continuous 96‐hour salt stress treatment (Figure 1F,G). Tissue‐specific expression analysis demonstrated that *MdWRKY33d* was expressed in all apple organs (root, stem, leaf), with significantly higher levels observed in roots and leaves compared to stems (Figure S3D). Subcellular localization experiments further validated *MdWRKY33d* as a transcription factor, with its green fluorescent protein (GFP)‐fused protein localizing exclusively to the nucleus (Figure S3C).

**Figure 1:**
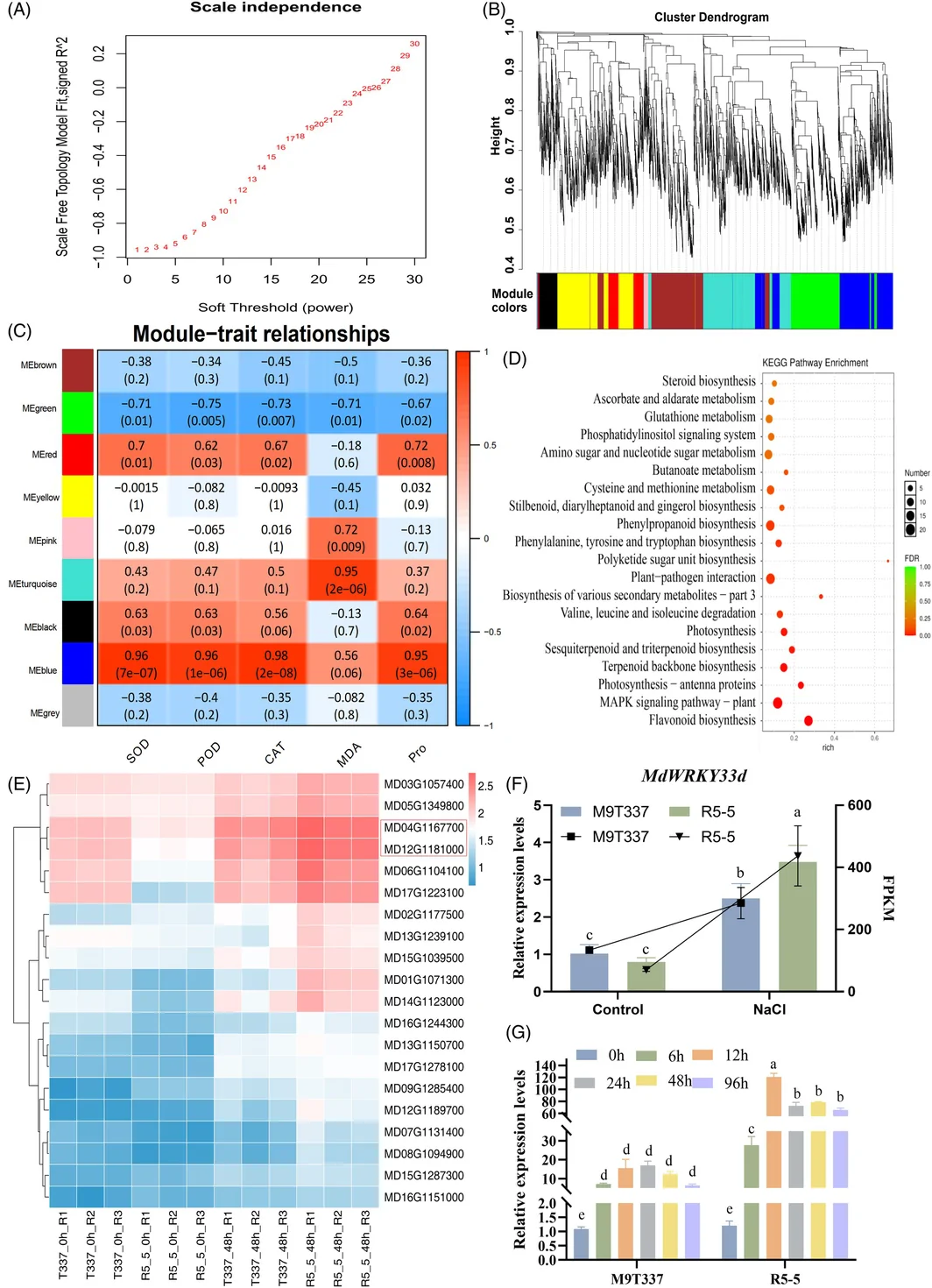
Weighted gene co‐expression network analysis of genes related to salt stress. (A) Soft power plot. The abscissa represents the soft threshold (β), and the ordinate represents the scale‐free topology model fit index. (B) Clustering dendrogram of genes and module division. (C) Correlations between modules and physiological indices by WGCNA analysis under control and salt stress treatments. The X‐axis represents physiological phenotypes; the Y‐axis represents different gene module hierarchical clusters. Numbers within modules indicate the correlation (significance) between modules and various physiological indices, with red indicating positive and blue indicating negative correlations. (D) KEGG enrichment analysis of MEblue module genes. (E) Differentially expressed analysis of WRKY genes of the MEblue module in the transcriptome of R5‐5 (salt‐resistant) and T337 (salt‐sensitive) roots under salt treatment. (F, G) Relative expression levels of MD04G1167700 in R5‐5 (salt‐resistant) and T337 (salt‐sensitive) under salt treatment. The error bars represent the SD of three biological replicates (*n* = 3). Statistical significance was determined by two‐way ANOVA followed by Tukey's multiple comparisons test (*P* < 0.05).

### *MdWRKY33d* enhances ROS clearance and maintains photosynthetic system stability in transgenic apple plants under salt stress

To further validate *MdWRKY33d* function in apple, transient transformation assays were conducted using M9T337 apple rootstock, obtaining pCAMBIA2300‐*WRKY33d* (*WRKY33d*‐OE), pCAMBIA2300, TRV2‐*WRKY33d* and TRV2 transgenic lines. RT‐qPCR was used to confirm the transformation and the up‐regulation or down‐regulation of *MdWRKY33d* expression in different transgenic lines (Figure 2B,F). After exposure to 150 mM NaCl for 2 and 4 days, *WRKY33d*‐OE plants exhibited milder symptoms of salt injury, while TRV2‐*WRKY33d* plants exhibited aggravated symptoms (Figure 2A,E). Physiological assays showed that *WRKY33d*‐OE plants had significantly lower MDA content and significantly higher proline content compared to the pCAMBIA2300 control (Figure 2C,D). However, TRV2‐*WRKY33d* plants exhibited the opposite pattern, with higher MDA and lower proline than TRV2 controls (Figure 2G,H). Furthermore, staining with 3,3′‐diaminobenzidine (DAB) and nitroblue tetrazolium (NBT) showed lower ROS accumulation in *WRKY33d*‐OE lines than in pCAMBIA2300 control lines, but higher ROS levels in TRV2‐*WRKY33d* lines than in TRV2 control lines (Figure 2I,O). Quantitative measurements of H_2_O_2_ and O_2_^−^ supported these staining results (Figure 2J,K and P,Q). This indicates that MdWRKY33d alleviates the degree of membrane damage in *WRKY33d*‐OE lines under salt stress. In addition, WGCNA revealed that the MEblue module displayed a highly significant positive correlation with SOD (r = 0.96), POD (r = 0.96), and CAT (r = 0.98) activity. Notably, *MdWRKY33d* is a member of this co‐expression module, which implies its potential role in ROS scavenging. Similarly, after salt treatment, analysis of antioxidant enzyme activities (SOD, POD, and CAT) revealed that the activities of these enzymes in *WRKY33d*‐OE plants were significantly higher than in pCAMBIA2300 control plants (Figure 2L–N). Conversely, the activities in TRV2‐*WRKY33*d plants were significantly lower than in TRV2 control plants (Figure 2R–T).

**Figure 2:**
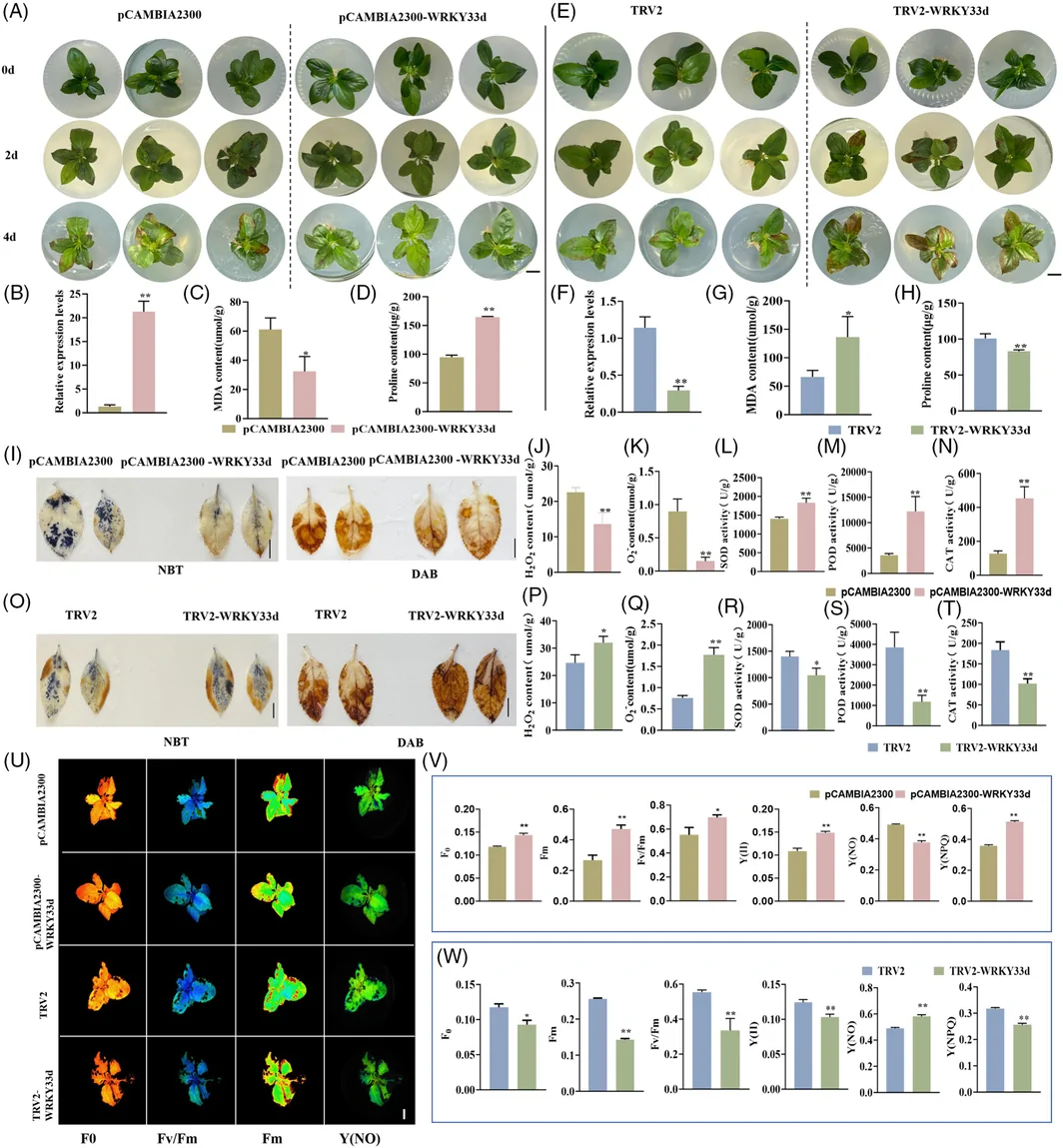
*MdWRKY33d* improves salt stress tolerance by scavenging ROS and maintaining photosystem II activity in transgenic apple plants under salt stress. (A–D) Morphological traits (A), relative expression level of *MdWRKY33d* (B), MDA content (C), and Proline content (D) in pCAMBIA2300‐*WRKY33d* and pCAMBIA2300 control plants after salt treatment. (E–H) Morphological traits (E), Relative expression level of *MdWRKY33d* (F), MDA content (G), and Proline content (H) of TRV2‐*WRKY33d* and TRV2 control plants after salt treatment. (I) NBT and DAB staining, (J) H_2_O_2_ content, (K) O_2_^−.^ content, (L) SOD activity, (M) POD activity, and (N) CAT activity of pCAMBIA2300‐*WRKY33d* and pCAMBIA2300 control plant after 150 mM salt treatment for 2 days. (O) NBT and DAB staining, (P) H_2_O_2_ content, (Q) O_2_^−.^ content, (R) SOD activity, (S) POD activity, and (T) CAT activity of TRV2‐*WRKY33d* and TRV2 control plants after 150 mM salt treatment for 2 days. (U) Chlorophyll fluorescence images, (V, W) F_0_ value, Fm value, Fv/Fm ratios, Y(II), Y(NPQ), and Y(NO) of pCAMBIA2300‐*WRKY33d*, TRV2‐*WRKY33d*, and pCAMBIA2300, TRV2 control apple plants under salt treatment. Error bars represent the SD of three biological replicates (*n* = 3). The experiments were repeated three times with similar results. Asterisks indicate significant differences (*P < 0.05, **P < 0.01) as determined by Student's t‐test.

In addition, salt stress impairs photosynthesis by inducing chlorophyll degradation, disrupting PSII structure, and inhibiting photosynthetic enzyme activity. This photodamage process is quantitatively assessable through chlorophyll fluorescence parameters, including significant declines in initial fluorescence (F_0_), maximum fluorescence (Fm), Yield of Photosystem II Y (II), Maximum Quantum Yield of PSII (Fv/Fm), and Yield of Regulated Non‐Photochemical Energy Dissipation Y (NPQ). Chlorophyll fluorescence imaging showed that the leaves of different lines had distinct fluorescence quenching features under salt stress. Compared to controls, PSII integrity was mostly kept in *WRKY33d*‐OE plants but severely damaged in TRV2‐*WRKY33d* plants (Figure 2U). Quantitative fluorescence parameter analysis revealed that, after salt‐stress treatment, the *WRKY33d*‐OE lines had significantly higher F_0_, Fm, Fv/Fm, Y(II), and Y(NPQ) values in the photoprotection system, along with a reduced Y(NO) from non‐photochemical pathways, compared to the pCAMBIA2300 control (Figure 2V). In contrast, TRV2‐*WRKY33d* lines showed the opposite trends compared to the TRV2 control (Figure 2W). These findings suggest that *MdWRKY33d* enhances plant salt tolerance by sustaining the core reactions of photosystem II (PSII) and optimizing energy allocation between photochemistry and photoprotection.

In conclusion, these research findings substantiate that *MdWRKY33d* enhances the salt tolerance of apples by scavenging ROS and optimizing the photosynthetic system.

### *MdWRKY33d* enhances ROS clearance and maintains photosynthetic system stability in transgenic poplar under salt stress

To elucidate the biological function of *MdWRKY33d* in salt stress response, transgenic ‘84 K’ poplar lines overexpressing *MdWRKY33d* (OE33d‐2 and OE33d‐6) were obtained (Figure S4). After 20 days of 150 mM NaCl treatment, wild‐type (WT) poplar seedlings exhibited typical salt stress symptoms, including inhibited growth and extensive leaf abscission. In contrast, the transgenic lines (OE33d‐2 and OE33d‐6) showed milder salt stress responses. By day 50, WT plants reached physiological mortality with apical necrosis and basal desiccation, whereas the transgenic lines retained only slight wilting and minimal leaf abscission (Figure 3A). For quantitative analysis, we statistically compared plant heights and root morphological parameters (surface area, volume, branching number, and length) between WT and transgenic lines after 50 days of salt stress. OE33d‐2 and OE33d‐6 lines showed 10 cm and 20 cm increases in plant height compared to WT (Figure 3C). Similarly, both transgenic lines also had larger root surface areas and root volumes, more root branching numbers, and longer root lengths (Figure 3D–G). To reduce environmental interference, WT and two transgenic lines (OE33d‐2, OE33d‐6) were also cultured in the same culture bottle containing 80 mM NaCl medium. Consistent with potted seedling observations, both transgenic lines exhibited greater plant heights, more developed root systems, and milder salt‐stress damage (Figure 3B). Materials for subsequent assays were collected from rooted plantlets grown on 80 mM NaCl medium *in vitro*.

**Figure 3:**
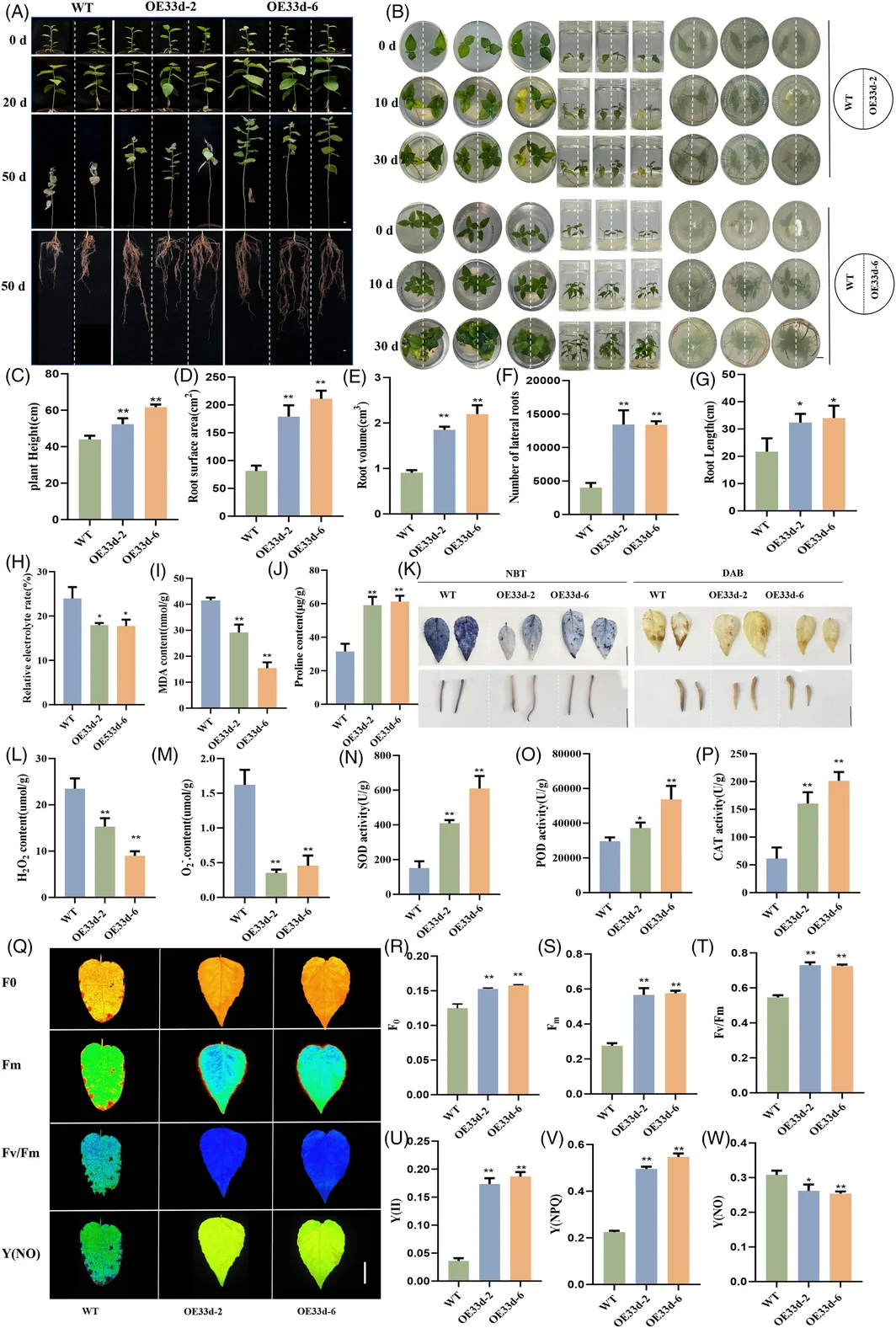
*MdWRKY33d* enhances poplar resistance to salt stress. (A) Representative phenotypic images of rooted seedlings from WT and MdWRKY33d‐overexpressing transgenic lines (OE33d‐2 and OE33d‐6) following transplantation into nutrient pots and subsequent exposure to 150 mM NaCl for 0 day (Control), 10 days, and 30 days. (B) Representative phenotypic images of WT and MdWRKY33d‐overexpressing transgenic lines (OE33d‐2 and OE33d‐6) were placed on rooting medium with 80 mM NaCl and treated for 0 day (Control), 10 days, and 30 days. (C–G) Measurements of plant height (C), root surface area (D), root volume (E), root branching number (F), and root length (G) of the indicated plants in (A). (H) REC, (I) MDA, (J) Proline content, (K) NBT and DAB staining, (L) H_2_O_2_ content, (M) O_2_^−^. content, (N) SOD activity, (O) POD activity, and (P) CAT activity of *MdWRKY33d*‐OE (OE33d‐2 and OE33d‐6) and WT after 150 mM salt treatment for 10 days. (Q) Chlorophyll fluorescence images, (R) F_0_ value, (S) Fm value, (T) Fv/Fm ratios, (U) Y(II), (V) Y(NPQ), and (W) Y(NO) of WT and *MdWRKY33d‐*OE poplar plants under salt treatment. Scale bar = 1 cm. Error bars represent SD from independent biological replicates (*n* = 6 for C‐G; *n* = 3 for all other panels). Asterisks indicate significant differences (*P < 0.05, **P < 0.01) as determined by Student's t‐test.

After salt stress, the *MdWRKY33d*‐OE lines showed significantly lower relative electrolyte leakage (REL) and MDA content, and significantly higher proline content compared to the WT (Figure 3H–J). Furthermore, we analyzed the accumulation of H_2_O_2_ and O_2_^−^ in the leaves and roots of WT and *MdWRKY33d*‐OE plants using DAB and NBT staining, respectively. The *MdWRKY33d*‐OE lines displayed fewer reddish‐brown block‐like spots and blue dot‐like spots compared to the WT (Figure 3K). The quantitative measurements of H_2_O_2_ and O_2_^−^ amounts were consistent with the staining results (Figure 3L,M). Moreover, the activities of SOD, POD, and CAT in OE33d‐2 and OE33d‐6 lines were significantly higher than those in the WT after salt stress (Figure 3N–P). These results indicate that *MdWRKY33d* improves the resistance of transgenic poplar to salt by enhancing antioxidant enzyme activity to clear ROS.

In addition, after salt stress, OE33d‐2 and OE33d‐6 leaves exhibited less fluorescence quenching compared to WT plants, indicating preserved PSII integrity (Figure 3Q). Quantitative analysis of fluorescence parameters showed that OE33d‐2 and OE33d‐6 lines maintained higher F_0_ and Fm, resulting in greater Fv/Fm than WT (Figure 3R–T). This enhanced photochemical efficiency correlated with higher Y(II) values in OE33d‐2 and OE33d‐6 under stress (Figure 3U), reflecting superior linear electron transport capacity. Additionally, compared to WT plants, transgenic lines showed reduced Y(NO) via non‐photochemical pathways and enhanced Y(NPQ) in the photoprotection system (Figure 3V,W). This coordinated regulatory mechanism effectively alleviated photoinhibition‐induced photodamage. These findings suggest *MdWRKY33d* enhances salt tolerance by maintaining PSII core reactions while optimizing energy allocation between photochemistry and photoprotection.

In conclusion, all the results consistently demonstrate that *MdWRKY33d* overexpression significantly improves the salt tolerance of transgenic poplars by scavenging ROS and optimizing the photosynthetic system.

### *MdWRKY33d* enhances ROS clearance in transgenic apple calli under salt stress

Additionally, *MdWRKY33d*‐OE and *MdWRKY33d*‐RNAi transgenic apple calli were generated to further investigate the function of *MdWRKY33d* under salt stress (Figure S5). The 15‐day‐old WT, *MdWRKY33d*‐OE, and *MdWRKY33d*‐RNAi transgenic calli were placed on MS medium containing 150 mM NaCl for 15 days and 25 days. The research findings demonstrated that the growth performance of *MdWRKY33d*‐OE transgenic calli was significantly superior to that of the WT, with an increase in fresh weight under salt stress conditions. Conversely, the growth of *MdWRKY33d*‐RNAi lines was significantly inhibited (Figure S6A). Compared with WT, *MdWRKY33d*‐OE calli exhibited significantly reduced MDA content and electrical conductivity, along with significantly elevated proline accumulation under salt stress, whereas *MdWRKY33d*‐RNAi calli showed opposing physiological trends (Figure S6B–E). DAB and NBT staining showed that under salt stress, ROS levels in *MdWRKY33d*‐OE were lower than in WT, while those in *MdWRKY33d*‐RNAi were higher. (Figure S6H). The quantitative determination results of H_2_O_2_ and O_2_^−^. contents were consistent with the staining results (Figure S6F,G). Furthermore, the activities of SOD, POD, and CAT in *MdWRKY33d*‐OE were significantly higher than those in the WT, whereas the activities of these three enzymes in *MdWRKY33d*‐RNAi were significantly lower (Figure S6I–K). Overall, *MdWRKY33d* overexpression improved salt tolerance in transgenic apple calli by activating antioxidant enzyme activity and improving ROS scavenging under salt stress.

### MdWRKY33d binds to promoter of *MdDREB1D* and *MdPER55* to activate their transcription

To gain the regulatory network of *MdWRKY33d* in salt stress responses, RNA‐seq analysis was performed using samples from WT and *MdWRKY33d*‐OE‐7 apple calli lines after salt stress. DEGs were identified using thresholds of |log2 (fold change)| > 2 and *P* < 0.05. Compared to WT, OE‐7 exhibited 1763 upregulated genes and 2677 downregulated genes under salt stress conditions (Figure S9A). KEGG enrichment analysis of these DEGs showed significant enrichment in the phenylpropanoid biosynthesis pathway (Figure S9B). The preceding results indicate that overexpressing *MdWRKY33d* can significantly enhance the salt tolerance of OE‐7 by scavenging ROS (Figure S6I–K). Therefore, we conducted an analysis of DEGs related to POD in the transcriptome and found that a peroxidase‐encoding gene (*MdPER55*) had significantly higher expression in OE33d‐7 lines compared to WT (Figure S9C). Moreover, in the transcriptome data of two rootstocks under salt stress, *MdDREB1D* showed the same expression trend as *MdWRKY33d* (Figure S7A,B). Furthermore, qRT‐PCR was used to measure the *MdDREB1D* and *MdPER55* expression levels in WT and *MdWRKY33d* transgenic lines under normal and salt stress conditions (Figures S8A,B and S9D,E). Under these conditions, both *MdDREB1D* and *MdPER55* were upregulated in the *MdWRKY33d*‐OE lines compared with the WT plants. By contrast, their expression was suppressed in TRV‐*MdWRKY33d* lines (Figures S8C and S9F). So, we speculate that they are likely direct target genes of *MdWRKY33d*.

To validate this, we further analyzed the promoter sequences of *MdPER55* and *MdDREB1D* and detected the W‐box motifs, respectively (Figure 4C,F). Subsequently, we carried out yeast one‐hybrid (Y1H), electrophoretic mobility shift assay (EMSA), Dual‐Luciferase (Dual‐LUC) reporter gene experiments, and chromatin immunoprecipitation‐qPCR assay (ChIP‐qPCR) both *in vitro* and *in vivo*. In Y1H assays, co‐transformed yeast cells containing the pGADT7 empty vector and pAbAi‐p*MdPER55/MdDREB1D* could not grow on ‐Leu medium with 300 ng/mL AbA; however, co‐transformed yeast cells containing pGADT7‐MdWRKY33d and pAbAi‐p*MdPER55/MdDREB1D* could grow on ‐Leu medium with 300 ng/mL AbA (Figure 4A,D). In addition, we generated firefly luciferase reporter constructs driven by the *MdPER55* and *MdDREB1D* promoters and effectors using *MdWRKY33d* (Figure 6E). The Dual‐LUC assay showed that co‐expression of pGreenII 62‐*MdWRKY33d* and pGreen‐p*MdPER55/*p*MdDREB1D* resulted in a significant increase in luminescence intensity compared with the empty vector and a raised LUC/REN ratio in *N. benthamiana* leaves (Figure 4B,E). The EMSA results demonstrated that MdWRKY33d bound to the W‐box sites of the *MdPER55* and *MdDREB1D* promoters, and the binding intensity gradually decreased as a gradient of competitor concentrations was added (Figure 4C,F). Subsequently, ChIP‐qPCR assays were performed to verify this interaction *in vivo*. The results showed that MdWRKY33d was significantly enriched at the W‐box motifs of the *MdPER55* and *MdDREB1D* promoters (Figure 4G). In summary, *MdWRKY33d* can directly bind to the *MdPER55* and *MdDREB1D* promoters and activate their transcription both *in vitro* and *in vivo*.

**Figure 4:**
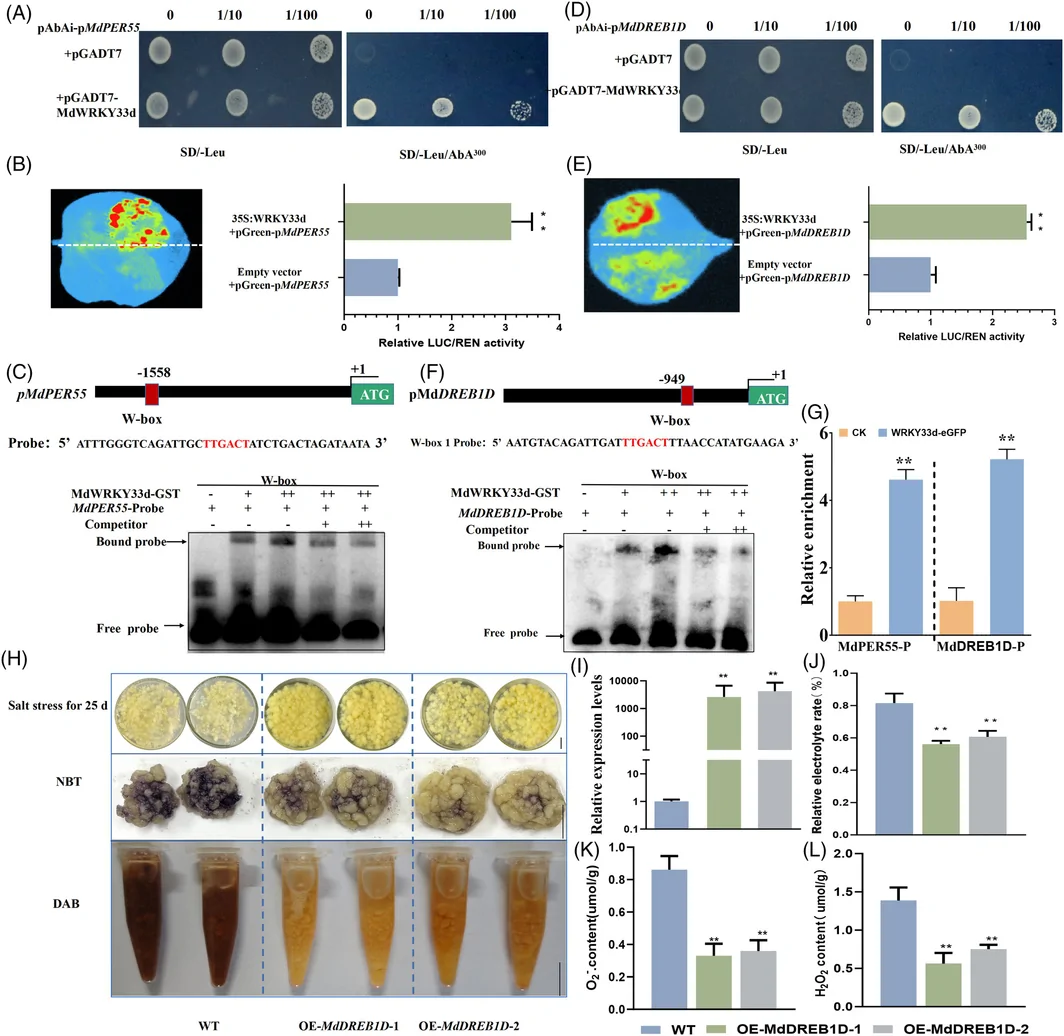
MdWRKY33d activates *MdPER55/MdDREB1D* by directly binding to their promoter region to enhance salt tolerance. (A, D) Y1H assays showing the binding of MdWRKY33d to the promoters of *MdPER55/MdDREB1D*. Negative control: pAbAi‐p*MdPER55/MdDREB1D* + pGADT7. All yeast cells grew on SD/−Leu medium with (right) or without (left) AbA. (B, E) Live imaging and quantitative analysis of transcriptional activation of the *MdPER55/MdDREB1D* promoter by MdWRKY33d in tobacco leaves using the Dual‐LUC system. The control is co‐transformed with the empty vector pGreenII 62‐SK and pGreen‐p*MdPER55/MdDREB1D*. (C, F) Schematic diagram of the *MdPER55/MdDREB1D* promoter regions containing potential MdWRKY33d binding sites, and EMSA assay of the specific binding of MdWRKY33d to W‐box elements in promoters of *MdPER55/MdDREB1D*. The purified MdWRKY33d‐GST protein and biotin‐labeled probes of designed fragments containing the TTGACT motif were used. (G) ChIP‐qPCR showing MdWRKY33d binding to the *MdPER55/MdDREB1D* promoter *in vivo*. Relative enrichment was normalized to the input and expressed as the ratio of the MdWRKY33d‐eGFP line to EV‐eGFP (set as 1.0). (H) *MdDREB1D* improves salt stress tolerance of apple calli by scavenging ROS. Morphological traits, NBT and DAB staining of MdDREB1D‐OE and WT after salt treatment (150 mM for 25 days). (I) *MdDREB1D* expression levels, (J) REC, (K) O_2_^−.^ content, (L) H_2_O_2_ content, in MdDREB1D‐OE and WT after 150 mM salt treatment for 25 days. Scale bar = 1 cm. Data represent the SD from three independent experiments (*n* = 3). Asterisks indicate significant differences (**P < 0.01**) as determined by Student's t‐test.

Under salt stress, the expression levels of *MdDREB1D* and *MdPER55* were significantly higher in R5‐5 than in M9T337, a trend that persisted throughout the 96 h treatment period (Figures S7C and S9H). Therefore, we hypothesize that these two genes contribute to salt tolerance in apple. To validate their roles, we successfully generated transgenic apple calli lines overexpressing *MdDREB1D* and *MdPER55*, respectively (Figure 4G,H, Figure S9I,J). Consistently, after 25 days of salt stress treatment, both the *MdDREB1D*‐OE and *MdPER55*‐OE transgenic apple calli displayed significantly higher tolerance to salt stress compared to the control. Notably, the electrolyte leakage rate, as well as the amounts of H_2_O_2_ and O_2_^−^, were markedly lower in the transgenic lines than in the control (Figure 4J,K, Figure S9K–M). Correspondingly, the POD enzymatic activity was significantly elevated in the *MdPER55*‐OE lines (Figure S9N). This finding suggests that *MdDREB1D* and *MdPER55* positively regulate salt tolerance in apples by improving ROS scavenging. Furthermore, we examined the expression of two genes in each other's overexpression lines and found that *MdDREB1D* expression remained unchanged in *MdPER55*‐OE lines (Figure S9O). In contrast, *MdPER55* expression was significantly upregulated in *MdDREB1D*‐OE lines compared to WT under both salt stress and control conditions, suggesting that *MdPER55* acts downstream of *MdDREB1D* (Figure S9G). However, no DRE/CRT (CCGAC) motifs were identified within the *MdPER55* promoter, implying that *MdDREB1D* indirectly modulates *MdPER55* expression, potentially through intermediate regulatory factors.

Collectively, these results demonstrate that MdWRKY33d directly binds to the *MdDREB1D* and *MdPER55* promoters and activates their transcription, thereby enhancing ROS‐scavenging capacity and salt tolerance in apple.

### MdVQ22 physically interacts with MdWRKY33d *in vitro* and *in vivo*

To explore the molecular mechanism of the MdWRKY33d regulated salt stress response in depth, a Y2H screening experiment was performed to identify MdWRKY33d‐interacting proteins. Full‐length MdWRKY33d exhibited transcriptional autoactivation when fused to the DNA‐binding domain (BD) of the pGBKT7 vector. To eliminate this background activity, N‐terminal truncated variants of MdWRKY33d (amino acids 71–520, 167–520, and 214–520) harboring dual WRKY domains were constructed in the pGBKT7 bait vector (Figure 5A). These variants were co‐transformed with the pGADT7 prey vector into Y2HGold competent yeast cells to evaluate transcriptional self‐activation. These data conclusively demonstrated the absence of transcriptional self‐activation in both the truncated bait construct pGBKT7‐MdWRKY33d^(167–520)^ and pGBKT7‐MdWRKY33d^(214–520)^ vectors (Figure 5A). Consequently, the bait vector pGBKT7‐MdWRKY33d^(167–520)^ containing the longest fragment (amino acids 167–520) was selected for subsequent yeast library screening. We isolated a VQ‐motif containing protein, MdVQ22, as an MdWRKY33d‐interacting protein (Figure 5B). The coding sequence (CDS) of *MdVQ22* is 756 bp and encodes a protein with 251 amino acids. Analysis of the amino acid sequence shows that MdVQ22 harbors the conserved domain FxxhVQxhTG and belongs to the VQ protein family (Figure S10A). A phylogenetic analysis indicated that MdVQ22 is closely related to PbVQ22 from pear (Figure S10B). Subcellular localization assays showed that MdVQ22 is localized in the nucleus (Figure S10C). The *MdVQ22* gene exhibited significantly higher expression in the roots compared to that in the stems and leaves (Figure S10D).

**Figure 5:**
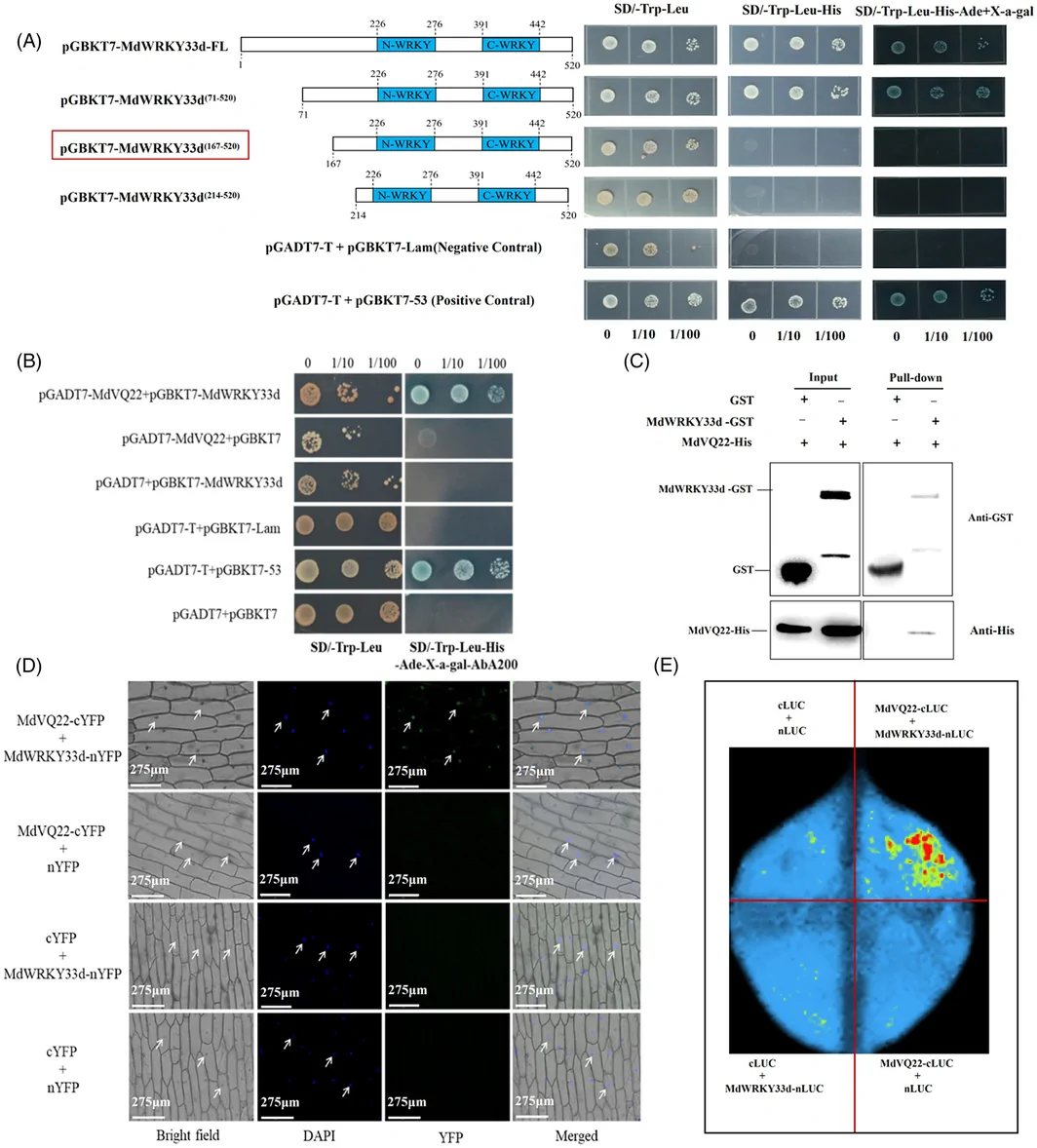
MdVQ22 physically interacts with MdWRKY33d *in vitro* and *in vivo*. (A) Schematic of MdWRKY33d domain architecture and transcriptional activation profiling of full‐length (1–520 aa) and N‐terminal truncated variants (71–520, 167–520, 214–520 aa). The blue boxes indicate the conserved WRKY domains. (B) Y2H assay to detect the interactions between MdWRKY33d^(167–520)^ and MdVQ22 in yeast cells. pGBKT7‐53 served as a positive control. Empty BD bait vector plus empty AD prey vector, empty BD bait vector plus AD‐MdVQ22 fusion prey vector, and pGBKT7‐Lam served as negative controls. The experiments were repeated three times with similar results. A representative photograph is shown here. (C) Pull‐down assay for detecting the interaction between MdVQ22 and MdWRKY33d. MdWRKY33d‐GST or purified GST was incubated with MdVQ22‐His protein and purified using a GST Purification Kit. Resultant protein samples were immunoblotted with anti‐GST or anti‐HIS antibodies. The negative control was GST. (D) Assay of bimolecular fluorescence complementation (BiFC), showing fluorescence in nuclear compartments of tobacco leaf epidermal cells that resulted from the complementation of the C‐terminus part of YFP fused to MdVQ22 (MdVQ22‐cYFP) with the N‐terminus part of YFP fused to MdWRKY33d (MdWRKY33d‐nYFP). No signal was observed from negative controls. Scale bar = 275 μm. (E) Split‐luciferase complementation assay showing the interaction of MdVQ22 and MdWRKY33d. The construct combinations of MdVQ22 and MdWRKY33d were co‐transformed into tobacco leaves.

Further, we performed pull‐down assays, bimolecular fluorescence complementation (BiFC), and luciferase complementation imaging (LCI) assays to verify the interaction between MdWRKY33d and MdVQ22 *in vivo* and *in vitro*. In pull‐down assays, the MdWRKY33d‐GST and MdVQ22‐His fusion proteins were mixed and co‐incubated with glutathione affinity gel resin, using purified GST protein as a negative control. Following elution with glutathione, protein complexes were detected by Western blotting (WB) using anti‐His and anti‐GST antibodies. The experimental results demonstrated that MdWRKY33d‐GST specifically captured the MdVQ22‐His protein, forming a stable protein complex, whereas the GST control protein failed to bind MdVQ22‐His (Figure 5C). In the BiFC assays, when MdWRKY33d‐nYFP was co‐infiltrated with MdVQ22‐cYFP in tobacco leaves, a YFP signal was detected in the nucleus; however, cYFP + MdWRKY33d‐nYFP, MdVQ22‐cYFP + nYFP, and nYFP + cYFP showed no fluorescence (Figure 5D). In the LCI assays, strong LUC activity was observed when MdVQ22‐cLuc was co‐injected with MdWRKY33d‐nLuc into tobacco leaves (Figure 5E). These observations suggest that MdVQ22 interacts with MdWRKY33d *in vivo* and *in vitro*.

### *MdVQ22* negatively regulates apple salt resistance by suppressing the transcriptional activation activity of *MdWRKY33d*

In this study, *MdVQ22* and *MdWRKY33d* exhibited a significant inverse correlation in their expression profiles under salt stress. (Figure 1G, Figure S10E). In the salt‐sensitive apple rootstock M9T337, *MdVQ22* transcript levels remained constitutively high throughout the entire 96‐hour treatment. Conversely, in the salt‐tolerant rootstock R5‐5, MdVQ22 expression shows a transient induction pattern, reaching a peak at 6 hours post‐salt stress before rapidly declining to pre‐stress basal levels (Figure S10E). This genotype‐specific divergence suggests that *MdVQ22* may function as a negative regulator. To delve deeply into its role, we successfully obtained two OE‐*MdVQ22* transgenic apple calli lines. Under salt stress, these lines exhibited more severe growth inhibition and significantly lower fresh weights compared to WT (Figure 6A,B, Figure S5C), confirming that *MdVQ22* negatively regulates salt tolerance in apple. Furthermore, the transient transformation assays were conducted using M9T337 apple rootstock, obtaining TRV2‐*VQ22* and TRV2 transgenic lines. RT‐qPCR was used to confirm the transformation and the down‐regulation of *MdVQ22* expression in TRV2‐*VQ22* transgenic lines (Figure 6F). Under salt stress for 2 and 4 days, TRV2‐*VQ22* plants exhibited milder salt injury symptoms compared to the TRV2 control lines (Figure 6E). Moreover, the quenching amplitude in TRV2‐*VQ22* leaves was much lower than in TRV2 control leaves under salt stress. The TRV2‐*VQ22* lines exhibited significantly higher F, Fm, Fv/Fm, Y(II), and Y(NPQ) values, along with a lower Y(NO) value compared to the TRV2 control (Figure S11). Here, histochemical staining and quantitative analysis revealed lower H_2_O_2_ and O_2_^−.^ contents in TRV2‐*VQ22* lines compared to TRV2 under salt stress (Figure 6J–L). Consistent with the reduced ROS levels, the activities of SOD, POD, and CAT were significantly higher in TRV2‐*VQ22* lines compared to TRV2 under salt stress (Figure 6M–O). This indicates that the silencing of *MdVQ22* in apples enhances the salt tolerance of TRV2‐*VQ22* transgenic lines by elevating peroxidase activity and reducing the accumulation of ROS. Previously, we confirmed the interaction between MdWRKY33d and MdVQ22 (Figure 5). To investigate whether MdVQ22 affects the regulatory function of *MdWRKY33d* on *MdPER55*/*MdDREB1D* transcription, we assessed *MdPER55* and *MdDREB1D* expression levels in transgenic lines. In normal and salt stress conditions, expression levels of both genes were significantly lower in OE‐*MdVQ22* apple calli lines compared to WT (Figure 6C,D). Conversely, their expression levels were significantly higher in TRV2‐*VQ22* transgenic lines compared to the TRV2 control (Figure 6G–I). EMSA assays also revealed that MdVQ22 significantly attenuated the binding of MdWRKY33d to *MdPER55* and *MdDREB1D* promoters (Figure 6P). Furthermore, transgenic apple calli co‐overexpressing MdVQ22 in the OE‐MdWRKY33d‐eGFP (OE33d‐7) background were generated and designated as OE‐(WRKY33d‐eGFP+MdVQ22). Consistently, ChIP‐qPCR confirmed *in vivo* that MdVQ22 inhibited the enrichment of MdWRKY33d at the W‐box sites within these promoters in the co‐expression lines (Figure 6Q). In addition, we also performed dual‐luciferase assays in tobacco leaves by co‐transforming combinations of pGreenII 62‐SK + Pro‐*MdPER55*/*MdDREB1D*‐LUC, MdVQ22 + Pro‐*MdPER55*/*MdDREB1D*‐LUC, MdWRKY33d + Pro‐*MdPER55*/*MdDREB1D*‐LUC, and MdVQ22 + MdWRKY33d + Pro‐*MdPER55*/*MdDREB1D*‐LUC into tobacco leaves (Figure 6R–T). The results showed that MdWRKY33d induced the activity of *MdPER55*/*MdDREB1D* transcripts, whereas the combination of MdVQ22 and MdWRKY33d significantly downregulated the transcription level of *MdPER55*/*MdDREB1D in vivo* (Figure 6R–T). Furthermore, phenotypic observations and fresh weight measurements revealed that the co‐overexpression of *MdWRKY33d* in the OE‐*MdVQ22*‐1 background partially rescued its salt‐sensitive phenotype (Figure 6U,V, Figure S5D). In normal and salt stress conditions, expression levels of both *MdPER55* and *MdDREB1D* were significantly higher in OE‐(*WRKY33d* + *MdVQ22*) apple calli lines compared to OE‐*MdVQ22* (Figure 6W,X). These findings all show that MdVQ22 negatively regulates apple salt resistance by inhibiting MdWRKY33d's capacity to transcriptionally activate *MdPER55*/*MdDREB1D*, thereby suppressing antioxidant enzyme activity and impairing ROS scavenging. In conclusion, this research uncovers the regulatory role of the MdVQ22‐MdWRKY33d‐*MdPER55/MdDREB1D* module in apple salt stress response (Figure 7).

**Figure 6:**
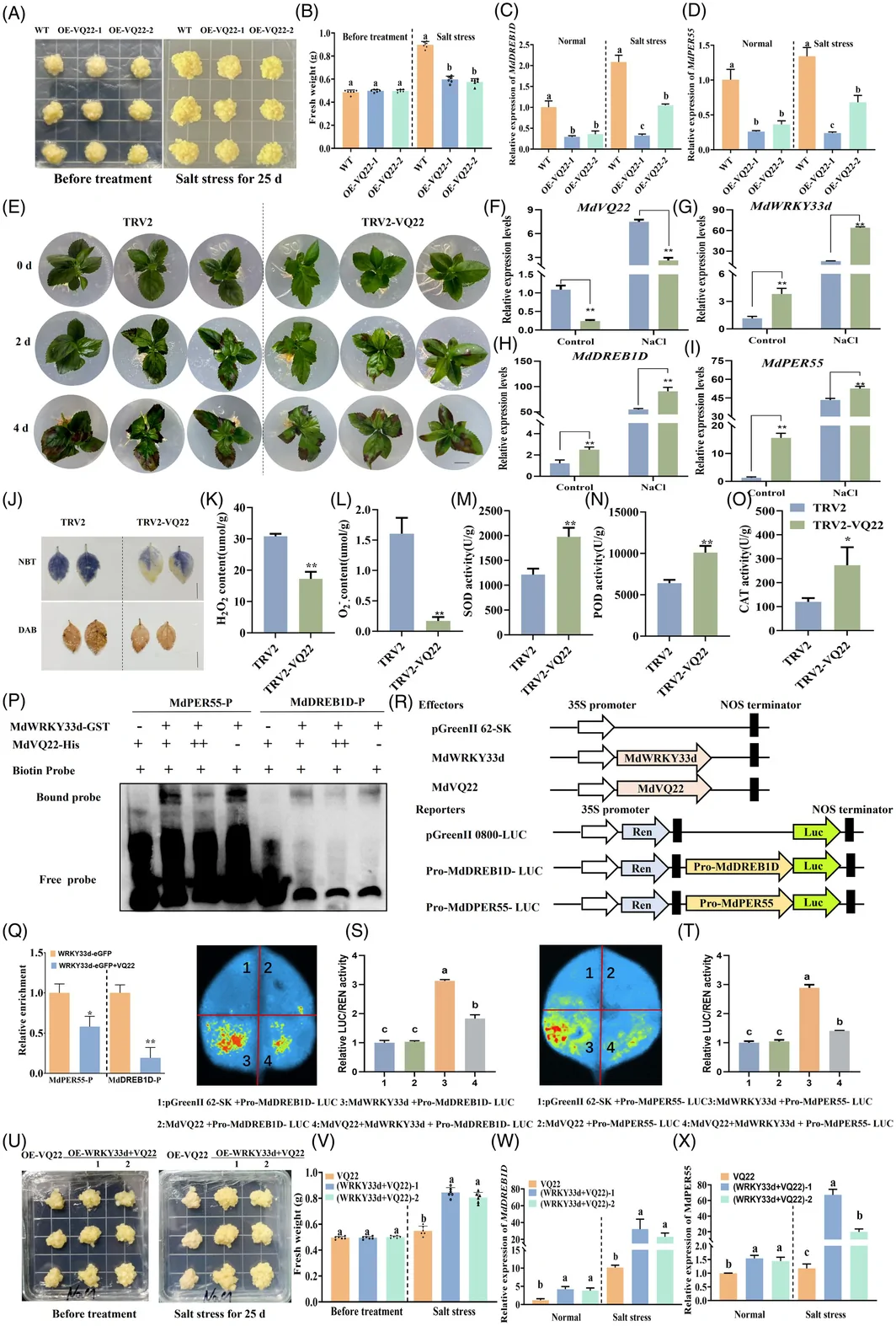
Overexpression of *MdVQ22* reduces the salt stress tolerance of apple by inhibiting the transcriptional activity of MdWRKY33d. (A) Morphological traits and (B) Fresh weight of *MdVQ22*‐OE apple calli under salt treatment (150 mM for 25 days). (C, D) Expression levels of *MdDREB1D* and *MdPER55* in MdVQ22‐OE apple calli lines. (E) Morphological traits and relative expression levels of *MdVQ22* (F), *MdWRKY33d* (G), *MdDREB1D* (H), and *MdPER55* (I) of TRV2‐*VQ22* and TRV2 control plants after salt treatment. (J) NBT and DAB staining, (K) H_2_O_2_ content, (L) O_2_^−^. content, (M) SOD activity, (N) POD activity, and (O) CAT activity of TRV2‐*MdVQ22* and TRV2 control plants after 150 mM salt treatment for 2 days. (P) EMSAs showing that MdVQ22 inhibits *MdWRKY33d* binding to the *MdDREB1D* and *MdPER55* promoters. (Q) ChIP‐qPCR assays showing that MdVQ22 inhibits MdWRKY33d binding to the *MdDREB1D* and *MdPER55* promoters. Relative enrichment was calculated as the fold change in OE‐(*MdVQ22* + *MdWRKY33d*) lines normalized to OE‐*MdWRKY33d*‐eGFP lines (set as 1.0). (R) Schematic of vector construction for transient expression assays. The *MdVQ22* and *MdWRKY33d* genes were cloned into the pGreenII62‐SK vector to generate the effector plasmids. The promoter fragments of *MdDREB1D* and *MdPER55* were recombined into pGreenII 0800‐LUC to create reporter plasmids. (S) Dual‐Luc reporter assay results and LUC activity of co‐expressed *MdVQ22*, *MdWRKY33d*, and Pro‐*MdDREB1D*‐luc. (T) Dual‐Luc reporter assay results and LUC activity of co‐expressed MdVQ22, MdWRKY33d, and Pro‐MdPER55‐luc. Different combinations of reporter and effector plasmids were co‐infiltrated into the leaves of *N. benthamiana*. Empty vector pGreenII 62‐SK was used as the reference. (U) Morphological traits and (V) Fresh weight of *MdVQ22*‐OE and *MdVQ22* + *MdWRKY33d*‐OE apple calli under salt treatment (150 mM for 25 days). (W, X) Expression levels of *MdDREB1D* and *MdPER55* in *MdVQ22*‐OE and *MdVQ22* + *MdWRKY33d*‐OE apple calli lines. Scale bar = 1 cm. Error bars represent SD from independent biological replicates (*n* = 6 for B and V; *n* = 3 for all other panels). Asterisks indicate significant differences (*P < 0.05, **P < 0.01) as determined by Student's t‐test. Different letters indicate significant differences between different combinations, according to one‐way ANOVA and Tukey's test (*P* < 0.05).

**Figure 7:**
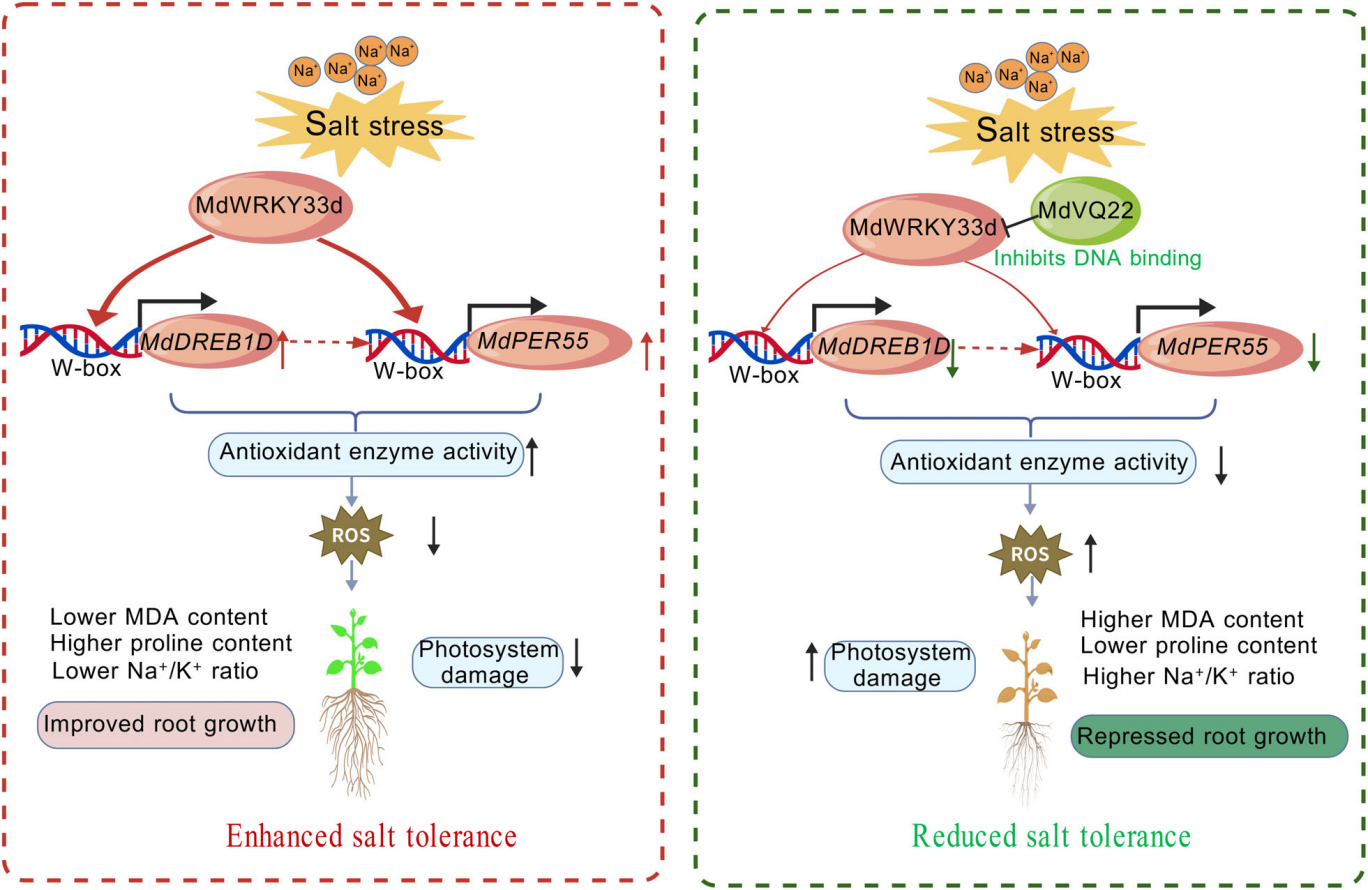
Model for MdVQ22‐MdWRKY33d‐*MdPER55/MdDREB1D* module‐mediated salt resistance in apple. Under salt stress, *MdWRKY33d* enhances ROS scavenging and maintains the photosynthetic system by directly binding to and activating the promoter region of *MdPER55* and *MdDREB1D*, thereby improving salt tolerance in both apple and poplar. Additionally, further investigation using Y2H screening identified MdVQ22 as a protein interacting with MdWRKY33d. Importantly, MdVQ22 negatively regulated salt tolerance in apple by suppressing the DNA‐binding activity of MdWRKY33d to the promoters of *MdPER55* and *MdDREB1D*.

To explore the conservation of this regulatory module in woody plants, we selected poplar as the model species for systematic validation. Sequence alignment revealed that the core functional domains of VQ22 and WRKY33 are highly conserved between apple and poplar (Figure S12RE,F). Time‐course expression analysis under salt stress showed that *PtVQ22*, *PtWRKY33*, *PtPER55*, and *PtDREB1* were all significantly induced, with consistent expression patterns to their apple orthologs (Figure S12A–D). Y2H assays confirmed the physical interaction between the poplar orthologs PtVQ22 and PtWRKY33, mirroring the mechanism observed in apple (Figure S12G). Heterologous overexpression of *MdWRKY33d* in poplar (OE33d‐2, OE33d‐6) significantly upregulated endogenous *PtPER55* and *PtDREB1* (Figure S12H–I), supporting the cross‐species functional compatibility of the WRKY33‐associated downstream response. Collectively, these results indicate that the VQ22‐WRKY33d‐*PER55/DREB1* regulatory relationship may be conserved between apple and poplar.

## DISCUSSION

### *MdWRKY33d* is a positive regulator in response to salt stress in apple

Soil salinization is now increasingly recognized as a major global environmental issue. Salt stress brings about significant negative impacts on apple trees, affecting their growth and development, fruit quality, and yield. Given that apples are primarily propagated via grafting, their stress resistance is largely determined by the rootstock. Moreover, roots are the first to be affected by adverse conditions such as salt stress, which subsequently inhibits the growth and development of above‐ground parts (Kawa et al., 2016). Under salt stress, roots enhance their ionic compartmentalization capacity by increasing lateral root density, expanding the root‐to‐shoot ratio, and forming salt‐exclusion channels (Hasegawa et al., 2000). Additionally, under salt stress, root damage can further suppress the growth and development of above‐ground parts (Kawa et al., 2016). For example, the accumulation of high concentrations of Na^+^ and Cl^−^ in chloroplasts under salt stress disrupts thylakoid membrane structure, thereby disturbing photosynthetic reactions (Chaves et al., 2009). Salt stress also disrupts photosynthetic metabolism, while indirectly exacerbating cellular damage through oxidative stress, ultimately compromising the photosynthetic capacity of leaves (Munns et al., 2006). Consequently, the root system traits of the rootstock directly govern the plant's tolerance to salt stress. In this study, there were no significant differences between the two rootstocks (R5‐5 and M9T337) under normal conditions. However, when subjected to salt stress, R5‐5 demonstrated higher photosynthetic efficiency, longer roots, a larger root surface area and volume, stronger root vitality, and a lower Na^+^/K^+^ ratio compared to M9T337 (Figures S1 and S2). These findings indicate that R5‐5 exhibits superior salt tolerance to M9T337.

To elucidate the physiological and molecular mechanisms underlying the different salt‐stress resistance of apple rootstocks, we performed physiological assays and transcriptomic sequencing on root samples from R5‐5 and M9T337, treated with 150 mM salt for 0 and 48 h. Transcriptome data analysis revealed that the WRKY genes within the MEblue module exhibited significant positive correlations with SOD, POD, and CAT activities as well as proline content (Figure 1A–C). Earlier studies have demonstrated that WRKY transcription factors play pivotal roles in plant responses to abiotic stresses (Yang et al., 2025). Currently, WRKY transcription factors associated with salt stress have been isolated and characterized in *Arabidopsis thaliana* (Krishnamurthy et al., 2020; Lu et al., 2023), rice (Yu et al., 2023), pineapple (Zhou et al., 2024), tomato (Shang et al., 2024), watermelon (Lan et al., 2025), Bok choy (Wang, Zheng, et al., 2025), and maize (Wang, Wei, et al., 2025). Herein, we successfully identified a salt‐responsive transcription factor, designated as *MdWRKY33d*. Notably, under salt stress, the up‐regulation level of *MdWRKY33d* in R5‐5 was significantly greater than that in M9T337 (Figure 1F), and it remained up‐regulated after 96 h exposure (Figure 1G). Furthermore, tissue‐specific expression analysis revealed that its expression levels in roots and leaves were higher than those in stems. Thus, we deduce that it might be involved in the regulation of apple salt stress tolerance. In fact, under salt stress, the OE‐MdWRKY33d transgenic poplar lines exhibited larger root surface areas and root volumes, a greater number of root branches, and longer root lengths compared to the WT plants (Figure 3). These results indicate that *MdWRKY33d* may enhance the salt tolerance of transgenic plants by regulating root development and morphological constitution.

The inhibition of the plant photosynthetic system caused by salt stress is a key factor influencing salt tolerance. Chlorophyll fluorescence parameters, acting as crucial indicators of the photosynthetic physiological state, are highly sensitive in reflecting damage to photosystem II (PSII) (Baker, 2008). In this study, *MdWRKY33d*‐overexpressing lines maintained significantly higher Fᵥ/F_m_ values under salt stress compared to WT. In contrast, *MdWRKY33d*‐silenced lines showed lower Fᵥ/F_m_ values under identical salt stress conditions (Figures 2 and 3, Figure S6). Also, salt stress induces damage to the cellular biological membrane, disrupting membrane system integrity. Elevated membrane permeability and the accumulation of lipid peroxidation products, such as MDA, are well‐established markers of membrane injury (Heath & Packer, 1968). The salt‐tolerant apple cultivars maintain cellular homeostasis by enhancing cell membrane stability, suppressing MDA accumulation, and promoting the biosynthesis of osmoprotective compounds like proline (Zhang, Li, et al., 2022). Simultaneously, salt stress disrupts the redox homeostasis of plant cells, leading to excessive accumulation of ROS and subsequent membrane lipid peroxidation and cellular dysfunction (Mittler, 2002). Plants mitigate oxidative damage by employing an antioxidant enzyme system composed of SOD, POD, and CAT to scavenge intracellular ROS (Gill & Tuteja, 2010). In this study, transgenic poplar and apple lines overexpressing *MdWRKY33d* accumulated less MDA than WT plants under salt stress, while TRV2‐silenced lines exhibited the opposite pattern (Figures 2 and 3; Figure S6). Moreover, salt treatment significantly elevated SOD, POD, and CAT activities in OE lines but reduced them in gene‐silenced lines (Figures 2 and 3; Figure S6). In summary, MdWRKY33d improves salt tolerance in transgenic poplar and apple by activating antioxidant systems and optimizing the photosynthetic system. However, the specific molecular mechanism of WRKY33d regulating plant response to salt stress remains to be fully elucidated.

### MdWRKY33d enhances apple salt tolerance by promoting the expression of the *MdPER55* and *MdDREB1D* genes

To gain insight into the biological processes downstream of *MdWRKY33d*, a comprehensive genome‐wide transcriptomic expression profiling analysis was performed using samples from WT and OE‐7 apple calli lines. The majority of peroxidase (PRXs) genes were upregulated in the OE‐7 lines compared with WT (Figure S9). As important ROS‐scavenging enzymes, peroxidases play a vital role in plant responses to environmental stresses. Overexpression of the peroxidase gene *ZmPRX1* increases maize seedling drought tolerance (Zhai et al., 2024). Overexpression of *TaPrx109‐B1* reduced leaf H_2_O_2_ levels and stomatal density, and increased tolerance to water deficit in wheat (Jiao et al., 2024). Overexpressing *TaPRX‐2A* in wheat also increased the tolerance of transgenic lines to salt stress (Su et al., 2020). Overexpression of *IbPRX17* promoted ROS scavenging and enhanced tolerance to salt and drought stress in sweet potato (Zhang, Wang, et al., 2022). In this study, we found that a peroxidase‐encoding gene (*MdPER55*) had significantly higher expression in the *MdWRKY33d* overexpression lines compared to WT. Moreover, previous research has shown that DREB1/CBF transcription factors are crucial regulators of salt tolerance in plants. The *AtDREB1F* (DDF1) gene responds to high salinity, and overexpressing *AtDDF1* enhances tolerance to high‐salinity stress in *Arabidopsis thaliana* (Magome et al., 2004, 2008). Overexpression of *OsDREB1D* enhances the high‐salt tolerance of transgenic Arabidopsis (Zhang et al., 2009). Additionally, *OsDREB1B* positively regulates salinity tolerance in rice seedlings (Liu et al., 2025). The GmDREB1B protein can bind to the promoter region of the *GmDof41* gene, thereby enhancing the salt and drought tolerance of soybeans (Kidokoro et al., 2015; Wei et al., 2023). In sweet cherry, the PaDREB1‐overexpressing transgenic plant exhibited greater salt tolerance than the wild‐type (Kitashiba et al., 2004). Consistent with previous findings, in our high‐salinity transcriptome data, we have also identified a homologous gene, *MdDREB1D*, which is located on a branch with a close evolutionary relationship to the Arabidopsis DREB1/CBF family (Figure S7). *MdDREB1D* exhibits a robust response to high‐salinity stress, and its induction level is significantly higher in resistant rootstock R5‐5 compared to sensitive rootstock M9T337 (Figure S7), showing the same expression trend as the *MdWRKY33d* gene (Figure S7A,B). Moreover, we generated *MdPER55* and *MdDREB1D* overexpression lines and discovered that they both positively regulate salt tolerance in apple (Figure 4H, Figure S9I). Further analysis of *MdPER55* and *MdDREB1D* promoter sequences revealed the presence of the W‐box motifs.(Figure 4C,F).

Notably, we found that both *MdPER55* and *MdDREB1D* were upregulated in *MdWRKY33d*‐OE lines compared to WT plants, while the opposite was observed in TRV‐*MdWRKY33d* lines under normal and salt stress conditions (Figure S8). Based on the findings above, we speculate that *MdPER55* and *MdDREB1D* are both likely direct target genes of *MdWRKY33d*. In fact, we validated this hypothesis using Y1H, EMSA, ChIP‐qPCR, and Dual‐Luc reporter experiments (Figure 4). In conclusion, MdWRKY33d can directly activate the transcription of *MdDREB1D* and *MdPER55* both *in vitro* and *in vivo*, thereby regulating salt tolerance in apple.

### MdVQ22 negatively regulates apple's salt resistance by suppressing the transcriptional activation activity of MdWRKY33d

WRKY TFs interact with various proteins to synergistically or antagonistically regulate plant growth and development, and respond to various stress responses. Many studies have shown that VQ proteins associate with WRKY TFs to form complexes that activate or inhibit WRKY transcriptional activity in apple. For example, MdVQ10 interacted with MdWRKY75 to enhance MdWRKY75‐activated transcription of *MdSAG12* and *MdSAG18* (Zhang et al., 2023). MdVQ37 inhibited the binding and transcriptional activity of *MdWRKY100* on the *MdPER3* and *MdRPM1* promoters (Dong et al., 2024; Duan et al., 2025). MdVQ17 can promote the binding and transcriptional activation activity of *MdWRKY17* on the promoter of *MdDMR6* (Duan et al., 2024). MdVQ12 promoted the transcriptional activity of *MdWRKY23* towards *MdHDA19* (Han et al., 2023). In apple, however, the interacting partners of MdWRKY33d in salt stress signaling remain largely uncharacterized. Herein, using Y2H screening, we isolated MdVQ22 as MdWRKY33d interacting proteins (Figure 5A,B). This physical interaction between MdVQ22 and MdWRKY33d was further validated by an *in vitro* pull‐down assay and an *in vivo* LCI assay (Figure 5C–E). The VQ proteins are involved in many aspects of plant growth and development as well as the biotic and abiotic stress responses. Previous studies have demonstrated that VQ‐WRKY complexes regulate salinity responses in several herbaceous species, including Arabidopsis, sweet potato, and tomato (Hu et al., 2013; Ma et al., 2023; Zhu et al., 2020). Nevertheless, the temporal dynamics, downstream regulatory relationships, and functional conservation of these modules in perennial woody species remain insufficiently understood. In the present study, *MdVQ22* and *MdWRKY33d* exhibited contrasting and genotype‐dependent expression patterns during salt stress (Figure S10E, Figure 1G). In salt‐sensitive rootstock M9T337, *MdVQ22* maintains sustained high expression levels; however, in salt‐tolerant rootstock R5‐5, its expression peaks at 6 h and rapidly returns to baseline (Figure S10E). This divergent pattern suggests that the rapid attenuation of *MdVQ22* in R5‐5 may be a prerequisite for activating salt defense mechanisms, whereas the inability of sensitive genotypes to downregulate *MdVQ22* leads to prolonged inhibition of the stress response. Consistently, downstream genes (*MdDREB1D* and *MdPER55*) were substantially up‐regulated in R5‐5, maintaining higher transcript levels than in M9T337 (Figures S7C and S9H), in agreement with the *MdWRKY33d* expression profile. Based on the observed interaction between MdVQ22 and MdWRKY33d (Figure 5C–E), we speculate that MdVQ22 antagonizes MdWRKY33d by forming inhibitory complexes, thereby hindering its recruitment to the promoters of salt‐tolerance genes. EMSA, ChIP‐qPCR, and dual‐LUC assays confirmed that *MdVQ22* acts as a negative regulator by suppressing the transcriptional activation capacity of MdWRKY33d on the *MdDREB1D* and *MdPER55* promoters (Figure 6E–R). As expected, overexpression of *MdDREB1D*, *MdPER55*, and *MdWRKY33d* significantly enhanced salt stress tolerance in transgenic lines by increasing POD activity to scavenge ROS (Figure 4, Figures S6 and S9). Conversely, *MdVQ22* overexpression significantly compromised salt tolerance in apple (Figure 6A). Notably, co‐overexpression of *MdVQ22* and *MdWRKY33d* in apple calli partially rescued the salt‐sensitive phenotype of the *MdVQ22*‐OE lines (Figure 6U). Interestingly, *MdPER55* was significantly up‐regulated in *MdDREB1D*‐OE lines relative to WT under both control and salt conditions, suggesting it functions downstream of *MdDREB1D* (Figure S9G). However, the absence of a canonical DRE/CRT motif (CCGAC) in the *MdPER55* promoter suggests that *MdDREB1D* may regulate *MdPER55* indirectly or *via* unidentified non‐canonical binding sites. Peroxidases (PRXs) play a pivotal role in abiotic stress response, primarily by regulating the antioxidant system. Studies have shown that some TFs can bind to PRX promoters to modulate their expression and subsequent POD activity, thereby enhancing abiotic stress tolerance in plants. For instance, the WOX11‐OsPRX130 module confers rice drought tolerance by maintaining ROS homeostasis in rice roots (Tan et al., 2025). The IbBBX24–IbTOE3–IbPRX17 module enhances abiotic stress tolerance by scavenging ROS in sweet potato (Zhang, Wang, et al., 2022). Here, we identified the VQ‐WRKY module involved in regulating *PRX*‐mediated H_2_O_2_ scavenging under salt stress conditions. Comparative analyses showed that the core functional domains of VQ22 and WRKY33 are conserved between apple and poplar. Y2H assays further confirmed a physical interaction between PtVQ22 and PtWRKY33. Moreover, heterologous overexpression of *MdWRKY33d* in poplar increased the expression of endogenous *PtPER55* and *PtDREB1* (Figure S12). These findings suggest a potentially conserved VQ22‐WRKY33‐*PER55*/*DREB1* regulatory relationship between apple and poplar, although broader conservation across perennial woody taxa requires further validation. In conclusion, this study reveals a mechanism for finely regulating salt stress resistance in apple. This mechanism is mediated by MdWRKY33d and its partner protein MdVQ22, functioning through the regulation of *MdDREB1D* and *MdPER55* transcription as well as other related stress‐responsive pathways (Figure 7). This finding provides a new perspective for a deeper understanding of the molecular mechanism of apple's response to salt stress and also offers potential molecular targets for the breeding of resistant apple varieties.

## MATERIALS AND METHODS

### Plant materials and salt treatments

The rooted tissue‐cultured seedlings of salt‐sensitive apple rootstock M9T337 and salt‐tolerant R5‐5 were grown in a 25°C greenhouse with 16 h light/8 h dark for 1 month. Afterward, uniformly growing plants were chosen and divided into normal and 150 mM NaCl treatment groups. Phenotypic observations and photos were taken on days 15, 20, and 25 post‐treatment. For 25 days treated materials, roots and mature leaves from both groups were collected for physiological analysis. Additionally, M9T337 and R5‐5 seedlings were hydroponically cultured, with root samples taken at 0, 6, 12, 24, 48, and 96 h post‐NaCl treatment for RNA extraction and RT‐qPCR. RNA‐seq was performed on samples from 0 and 48 h.

The calli of apple ‘Orin’ and transgenic apple calli lines were subcultured on MS medium with 0.225 mg/L 6‐BA and 1 mg/L 2,4‐D at 25°C in the dark. After 15 days, calli samples of wild‐type (WT) and transgenic lines with the same weight were placed on MS medium containing 150 mM NaCl for 25 days, and then physiological indicators were measured.

Rooted 84 K (*P. alba* × *P. glandulosa*) and transgenic poplar tissue‐cultured seedlings were moved to a 25°C greenhouse with a 16 h light/8 h dark cycle. After 2 weeks, they received 150 mM NaCl salt stress. On days 20 and 50 post‐stress, plant phenotypes were observed in detail, and photos were archived. Materials from day 20 of stress were used for physio‐biochemical tests. On day 50, root phenotypes were observed, and relevant data were measured.

To avoid environmental interference, when 84 K and transgenic poplar had 3–4 leaves, they were put into 1/2MS medium with 80 mM NaCl. On 0 days, 10 days, and 30 days post‐treatment, plant phenotypes were observed and photos taken. Also, apical mature leaves and new root tips from day 10‐treated materials were collected for ROS staining.

The transiently transformed transgenic apple seedlings were transplanted into MS medium in a growth chamber and kept in the dark for the initial 3 days. Subsequently, qRT‐PCR was employed to analyze the expression of target genes in the infected plants. After that, the transgenic apple rootstocks were transferred to a medium containing 150 mM NaCl. Phenotypic observations were conducted 2 days and 4 days after the treatment, respectively. Samples were collected 4 days later for the determination of physiological and biochemical indicators as well as gene expression analysis.

### Determination of physiological and biochemical characterization

After salt treatment, excised poplar and apple leaves, and apple calli from transgenic and WT plants were stained with nitroblue tetrazolium (NBT) to detect superoxide anion (O_2_^−^) accumulation and with 3,3′‐diaminobenzidine (DAB) to detect hydrogen peroxide (H_2_O_2_) accumulation, as described previously (Hou et al., 2018). Additionally, the activities of superoxide dismutase (SOD), peroxidase (POD), and catalase (CAT) were determined using detection kits from Solarbio (Solarbio, Beijing, China). Moreover, the contents of malondialdehyde (MDA), proline (Pro), H_2_O_2_, and O_2_^−^ were also measured using assay kits according to the manufacturer's instructions (Solarbio, Beijing, China). The electrolyte leakage was measured as previously described (Hou et al., 2018).

### Plasmid construction and plant genetic transformation

First, we amplified the coding sequence (CDS) of *MdWRKY33d*, *MdVQ22*, and *MdDREB1D* from R5‐5. To obtain overexpressing apple calli lines of *MdWRKY33d*, *MdVQ22*, *MdDREB1D*, and *MdPER55*, their CDS were fused into the pCAMBIA2300‐eGFP overexpression vector and verified by sequencing. To obtain transgenic apple calli lines of *MdWRKY33d*‐RNAi, Gateway technology was employed to fuse a 200 bp non‐conserved fragment from MdWRKY33d into the pK7‐GWIWG2D(II) vector, thereby constructing the *MdWRKY33d*‐RNAi vector. The constructed vectors were introduced into *Agrobacterium tumefaciens* strain ‘GV3101’ and transformed into apple ‘Orin’ calli (Chen et al., 2023). The transformed apple ‘Orin’ calli were spread on MS agar medium supplemented with 60 mg L^−1^ kanamycin to select positive lines. The positive lines were transferred to a new medium containing kanamycin to further remove the false positive material. Next, transgenic plants were confirmed by PCR with vector‐ and gene‐specific primers.

Furthermore, to obtain co‐expression transgenic lines, the CDS of *MdWRKY33d* and *MdVQ22* were separately cloned into the pCAMBIA1300 overexpression vector carrying a hygromycin resistance gene. After sequence verification, the resulting constructs were transformed into apple calli of the OE‐*MdWRKY33d*‐eGFP (OE33d‐7) or OE‐*MdVQ22* backgrounds by *Agrobacterium*‐mediated transformation. Positive co‐transformed lines were ultimately obtained through antibiotic selection on MS medium supplemented with 60 mg L^−1^ kanamycin and 10 mg L^−1^ hygromycin B.

To generate *MdWRKY33d* overexpressing poplar lines, *A. tumefaciens* strain ‘GV3101’ containing the pCAMBIA2300‐*MdWRKY33d* construct was used to transform poplar 84 K *via* the leaf disc method. Transgenic plants were confirmed through both kanamycin resistance screening and PCR analysis using vector and gene‐specific primers.

For virus‐induced gene silencing (VIGS), a nonconserved 350‐bp fragment of *MdWRKY33d* was inserted into the pTRV2 vector. The empty pTRV1, pTRV2, and pTRV2‐*MdWRKY33d* vectors were separately introduced into *A. tumefaciens* strain ‘GV3101’. The pTRV1 bacterial suspension was combined with the pTRV2‐*MdWRKY33d* or pTRV2 suspension and then infiltrated into 3‐week‐old tissue culture apple seedlings M9T337 through vacuum agroinfiltration (80 kPa, 20 min). The infiltrated plantlets were rinsed with distilled water, transplanted into MS medium in a growth chamber, and kept in the dark for the first 3 days. Then, qRT‐PCR was employed to analyze the expression of target genes in the infiltrated plants.

The primers used for vector construction are listed in Table S1.

### Quantitative RT‐PCR

Total RNA was extracted using the RNA prep Pure Plant Kit (Tiangen, Beijing, China), and reverse transcription was carried out with the PrimeScript First‐Strand cDNA Synthesis Kit (Takara, Otsu, Japan). qRT‐PCR experiments were done using SYBR Green Mix (TaKaRa, Otsu, Japan) on a Bio‐Rad CFX96 qPCR instrument (Bio‐Rad, Hercules, CA, USA) according to the manufacturer's instructions. The relative expression levels of genes were calculated using the 2^−ΔΔCT^ method with *MdActin* as the reference. All primers employed in the qRT‐PCR assays are listed in Table S1.

### Determination of chlorophyll fluorescence parameters

Leaves were placed in dark conditions for 20 min, and a FluorCam FC800 system (Photon Systems Instruments) was used to photograph the chlorophyll fluorescence (Deng et al., 2013). The minimum fluorescence (F0), maximum fluorescence (Fm), maximum photochemical efficiency of PSII photochemistry (Fv/Fm), actual photosynthetic efficiency of PSII Y(II), quantum yield of nonregulated energy dissipation Y(NO), and quantum yield of regulated energy dissipation Y(NPQ) were measured using a DualPAM‐100 chlorophyll fluorometer.

### Subcellular localization

The CDSs without the stop codons of *MdWRKY33d* and *MdVQ22* were ligated to a pCAMBIA2300‐eGFP vector to generate the MdWRKY33d/MdVQ22‐eGFP recombinant vector. *A. tumefaciens* strain ‘GV3101’ cells harboring this construct were bombarded into onion epidermal cells. Subcellular localization assays were carried out according to the method previously reported (Hou et al., 2018). The experiments were performed three times with similar results.

### Yeast one‐hybrid assays (Y1H)

Y1H assay was performed according to the manufacturer's instructions (Clontech Laboratories, Mountain View, CA, USA). The CDS of *MdWRKY33d* was inserted into the pGADT7 vector. The promoter of *MdDREB1D* and *MdPER55* was inserted into the pAbAi vector. The recombinant plasmids were transformed into the yeast strain Y1H. Transformed yeast cells were cultured in yeast synthetic dropout medium lacking Leu (SD/Leu) and 300 ng/mL AbA to confirm positive interactions. Empty pGADT7 vector served as a negative control.

### Electrophoretic mobility shift assay (EMSA)

The full‐length CDS of *MdWRKY33d* was introduced into the pGEX‐4T‐1 vector to generate a GST fusion protein. The correctly constructed GST‐MdWRKY33d vector was introduced into *Escherichia coli* BL21 (DE3) to express the recombinant protein, which was then purified by GST BindTM Resin (EMD Millipore Corp, USA) and identified by SDS‐PAGE and Western blotting. The sequence of the *MdDREB1D* and *MdPER55* promoters was analyzed, and the potential binding sites of MdWRKY33d, the W‐box [(C/T)TGAC(C/T)], were determined, and the corresponding EMSA biotinylated probes were designed. EMSA was conducted following the instructions of the Chemiluminescent EMSA Kit (Thermo Fisher Scientific, Waltham, Massachusetts, USA). The bands were scanned using TOUCH IMAGER (E‐BLOT, Shanghai, China). The probes used for EMSA are listed in Table S1.

### Dual‐luciferase transient expression assay (Dual‐LUC)

The full‐length CDS of *MdWRKY33d* and *MdVQ22* were cloned into the pGreenII 62‐SK vector, and the *MdDREB1D* promoter was inserted into the pGreenII 0800‐LUC vector and fused with the LUC reporter. The empty pGreenII 62‐SK vector was used as a negative control. These vectors were transformed into *A. tumefaciens* strain ‘GV3101’ and injected into tobacco leaves in different combinations. The infiltrated tobacco plants were cultured under a light–dark cycle in a growth chamber at 21°C for 3 days and then placed in a live fluorescence imager (VILBER LOURMAT, Paris, France) to observe fluorescence. The firefly luciferase (LUC) and Renilla luciferase (REN) were detected using the Dual‐Luciferase Reporter Gene Assay Kit (Vazyme, NanJing, China). Transcriptional activation of the target promoters was calculated as the ratio of LUC to REN.

### Yeast two‐hybrid assays (Y2H)

For the Y2H screening, an apple cDNA library was constructed by OE Biotech (Shanghai, China). The non‐self‐activating bait vector pGBKT7‐MdWRKY33d (167–520) was selected for library screening. Yeast transformation and screening were carried out according to the manufacturer's instructions (Clontech Laboratories, Mountain View, CA, USA). For targeted Y2H assays, the non‐self‐activating bait vector pGBKT7‐MdWRKY33d (167–520) was selected, and MdVQ22 was constructed on the activation domain (AD) of the pGADT7 vector. The AD‐ and BD‐expression vectors were transformed into Y2H yeast competent cells, plated on SD/−trp‐leu medium, and cultured at 30°C for 3 days. Individual colonies were selected and diluted with water to 1/10 and 1/100 dilutions. In SD/‐Trp‐Leu and SD/‐Trp‐Leu‐His‐Ade/X‐alpha‐gal/200 ng/mL AbA media, pGBKT7‐53 + pGADT7‐T was used as the positive control, and pGBKT7‐Lam + pGADT7‐T, pGBKT7 + pGADT7‐MdVQ22, pGBKT7‐MdWRKY33d (167–520) + pGADT7 and pGBKT7 + pGADT7 were used as the negative control.

### Bimolecular fluorescence complementation assays (BiFC)

The CDS of MdWRKY33d and MdVQ22 were amplified by PCR and inserted into pSPYNE‐35S and pSPYCE‐35S, respectively. The obtained fusion vectors, empty pSPYNE‐35S vector, and empty pSPYCE‐35S vector were transformed into *A. tumefaciens* strain ‘GV3101’ and co‐injected into tobacco leaves in different combinations. After 48 h of infection, yellow fluorescence in the infected leaves was observed using a confocal laser scanning microscope (LSM510 META; Zeiss, Oberkochen, Germany). 4′,6‐diamidino‐2‐phenylindole (DAPI) was used to stain cell nuclei.

### Split‐luciferase complementation assay (LCI)

The full‐length CDS of *MdWRKY33d* was cloned into the pCAMBIA1300‐nLUC vector, and *MdVQ22* was inserted into the pCAMBIA1300‐cLUC vector. The obtained fusion vectors, empty pCAMBIA1300‐nLuc vector, and empty pCAMBIA1300‐cLuc vector were transformed into *A. tumefaciens* strain ‘GV3101’. The tobacco was injected in accordance with the combinations of MdWRKY33d‐nLUC + MdVQ22‐cLUC, MdWRKY33d‐nLUC + cLUC, nLUC + MdVQ22‐cLUC, and nLUC + cLUC. The injected tobacco plants were cultured under light–dark cycles in a growth chamber at 21°C for 3 days. The fluorescence signal in the infected leaves was observed on a live fluorescence imager (VILBER LOURMAT, Paris, France).

### Pull‐down assay

MdWRKY33d‐GST fusion protein was obtained as described for the EMSA assay. MdVQ22‐His fusion proteins were produced in Escherichia coli strain BL21 (DE3) and purified using HIS purification columns (Beyotime, Shanghai, China). Protein mixtures were captured using the GST BindTM Resin (EMD Millipore Corp, USA). Eluted proteins were detected using anti‐GST and anti‐HIS antibodies.

### Chromatin immunoprecipitation‐qPCR assay (ChIP‐qPCR)

ChIP‐qPCR assays were performed as previously described (Jiang et al., 2020). MdWRKY33d‐GFP and MdVQ22 + MdWRKY33d‐GFP transgenic calli were treated with 150 mM NaCl for 12 h, then harvested and cross‐linked. After sonication, the chromatin was immunoprecipitated with an anti‐GFP antibody (Beyotime Biotechnology, Shanghai, China). Relative enrichment was quantified by qPCR, normalized to controls (set as 1.0). The primers are listed in Table S1.

### Statistical analyses

All data were analyzed using IBM SPSS (version 20) statistical software (IBM, Chicago, IL, USA). All data are presented as mean standard deviation (SD). Specific statistical tests are detailed in the corresponding figure legends. For comparisons across three or more groups, one‐way or two‐way analysis of variance (ANOVA) was performed, followed by Tukey's Honestly Significant Difference (HSD) post‐hoc test for pairwise multiple comparisons. For comparisons between two independent groups, an unpaired Student's t‐test was used. Statistical significance was defined as *P* < 0.05, and *P* < 0.01 was considered highly significant.

## AUTHOR CONTRIBUTIONS

HMH, SHJ and YGZ initiated and designed the study. HMH, QZ, GC, WGW, XLL, SXL, DCM, TAK and FMI performed the experiments. HMH, SHJ, XL and YGZ contributed the reagents/materials/analysis tools. HMH, SHJ, XL and YGZ contributed to resources and administration. HMH, SXL and QZ drafted the paper. HMH, SHJ and YGZ were involved in finalizing the manuscript.

## CONFLICT OF INTEREST

The authors declare no conflict of interest.

## Supporting information


**Figure S1.** Phenotypic and physiological changes in two apple rootstock varieties under normal conditions and salt stress. (A) Representative phenotypic images of salt‐sensitive rootstock M9T337 and salt‐tolerant rootstock R5‐5 at 15 days, 20 days, and 25 days under normal conditions and 150 mM NaCl. Scale bar = 1 cm. (B) The net photosynthetic rate (An), (C) transpiration rate (E), (D) stomatal conductance, and (E) intercellular carbon dioxide concentration (Ci) of both M9T337 and R5‐5 under normal conditions and 150 mM NaCl at 25 days. (F) Contents of K^+^, (G) Na+, and (H) the Na^+^/K^+^ ratio in the shoots of M9T337 and R5‐5 under normal conditions and 150 mM NaCl at 25 days. Error bars represent SD of three biological replicates (*n* = 3). Significant differences were analyzed by ANOVA with Duncan's multiple range test (*P* < 0.05) and are indicated with different letters.
**Figure S2.** Phenotypic and physiological changes in the roots of two apple rootstock varieties under normal conditions and salt stress. (A) Representative phenotypic images of the roots of salt‐sensitive rootstock M9T337 and salt‐tolerant rootstock R5‐5 at 25 days under normal conditions and 150 mM NaCl, respectively. Scale bar =1 cm. (B–E) Measurement of root volume (B), root surface area (C), root length (D), and root activity (E) of two apple rootstock varieties under normal conditions and salt stress. (F–J) REC (F), contents of MDA (G), K^+^ (H), and Na^+^ (I), and the Na^+^/K^+^ ratio (J) in the roots of M9T337 and R5‐5 under normal conditions and 150 mM NaCl at 25 days. Error bars represent the SD of three biological replicates (*n* = 3). Significant differences were analyzed by ANOVA with Duncan's multiple range test (*P* < 0.05) and are indicated with different letters.
**Figure S3.** Sequence analysis, phylogenetic analysis, tissue‐specific expression, and subcellular localization of apple MdWRKY33d. (A) Phylogenetic analysis of MD12G1181000 and MD04G1167700 and their homologous genes in Arabidopsis thaliana. The scale bar represents the replacement frequency of each amino acid site in the genome. (B) Amino acid sequence alignment of apple MD12G1181000 and MD04G1167700 and their homologous genes in other species. The red box represents the WRKYGQK domain. At, *Arabidopsis thaliana*; Pb, *Pyrus bretschneideri*; Os, *Oryza sativa*; Vv, *Vitis vinifera*. (C) Subcellular localization of apple MdWRKY33d. Bars = 125 μm. (d) Tissue‐specific expression analysis of *MdWRKY33d* in apple rootstock R5‐5. Error bars represent SD of three biological replicates (*n* = 3). Different letters indicate significant differences between different tissues, according to one‐way ANOVA and Duncan's test (*P* < 0.05).
**Figure S4.** Relative expression levels of *MdWRKY33d* in 84 K and transgenic lines. Error bars represent SD of three biological replicates (*n* = 3). Asterisks indicate significant differences (**P < 0.01) as determined by Student's t‐test.
**Figure S5.** Relative expression levels of *MdWRKY33d* in WT and transgenic apple calli lines. Error bars represent SD of three biological replicates (*n* = 3). Asterisks indicate significant differences (**P < 0.01) as determined by Student's t‐test.
**Figure S6.** MdWRKY33d improves salt stress tolerance of transgenic apple calli by scavenging ROS. (A) Morphological traits of MdWRKY33d‐OE (OE33d‐3 and OE33d‐7), *MdWRKY33d*‐RNAi (RNAi33d‐13 and RNAi33d‐15), and WT before and after salt treatment (150 mM for 20d and 25 days). (B) Fresh weight, (C) REC, (D) MDA, (E) Proline content, (F) H_2_O_2_ content, (G) O^2−^. content, (H) NBT and DAB staining, (I) SOD activity, (J) POD activity and (K) CAT activity of MdWRKY33d‐OE (OE33d‐3 and OE33d‐7), *MdWRKY33d*‐RNAi (RNAi33d‐13 and RNAi33d‐15), and WT after 150 mM salt treatment for 25 days. Error bars represent SD from independent biological replicates (*n* = 6 for B; *n* = 3 for all other panels). Different letters indicate significant differences between different tissues, according to one‐way ANOVA and Tukey's test (*P* < 0.05).
**Figure S7.** (A) Differentially expressed analysis of *DREB* genes in the transcriptome of R5‐5 (salt‐resistant) and M9T337 (salt‐sensitive) roots salt treatment. (B) Phylogenetic analysis of *MdDREB1D* and its homologous genes in *Arabidopsis thaliana*. The scale bar represents the replacement frequency of each amino acid site in the genome. (C) Relative expression levels of *MdDREB1D* in R5‐5 and M9T337 under salt treatment. The error bars represent the SD of three biological replicates (*n* = 3). Statistical significance was determined by two‐way ANOVA followed by Tukey's multiple comparisons test (*P* < 0.05).
**Figure S8.** The expression levels of the *MdDREB1D* gene in control plants and transgenic lines under both normal and salt‐stress conditions. (A) *MdWRKY33d* overexpression transgenic apple calli and WT. (B) pCAMBIA2300‐*WRKY33d* and pCAMBIA2300 transgenic apple seedlings. (C) TRV2‐*WRKY33d* and TRV2 transgenic apple seedlings. The error bars represent the SD of three independent experiments (*n* = 3). Asterisks indicate significant differences (*P < 0.05, **P < 0.01) as determined by Student's t‐test.
**Figure S9.** *MdPER55* improves salt stress tolerance of transgenic apple calli by scavenging ROS. (A) Volcano plots of differentially expressed genes. differential genes for *MdWRKY33d* transgenic apple calli OE‐7 and WT. (*P* < 0.05 and |log2(fold change)| > 2). (B) KEGG analysis showing differential gene enrichment terms for OE‐7 and WT. (C) Differentially expressed analysis of *PER* genes in the transcriptome of *MdWRKY33d* transgenic apple calli OE‐7 and WT (*P* < 0.05 and |log2(fold change)| > 2). (D) Relative expression of *MdPER55* in *MdWRKY33d* overexpression transgenic apple calli and WT. (E) Relative expression of *MdPER55* in pCAMBIA2300‐*WRKY33d* and pCAMBIA2300 transgenic apple seedlings. (F) Relative expression of *MdPER55* in TRV2‐*WRKY33d* and TRV2 transgenic apple seedlings. (G) Relative expression of *MdPER55* in *MdDREB1D* overexpression transgenic apple calli and WT. (H) Relative expression levels of *MdPER55* in R5‐5 and M9T337 under salt treatment. (I) *MdPER55* improves salt stress tolerance of apple calli. Scale bar = 1 cm. (J) *MdPER55* expression levels, (K) REC, (L) O_2_^−^. content, (M) H_2_O_2_ content, (N) POD activity in *MdPER55*‐OE and WT after 150 mM salt treatment for 25 days. (O) Relative expression of *MdDREB1D* in *MdPER55* overexpression transgenic apple calli and WT. The error bars represent the SD of three independent biological experiments (*n* = 3). Asterisks indicate significant differences (*P < 0.05, **P < 0.01) as determined by Student's t‐test. For (H), statistical significance was determined by two‐way ANOVA followed by Tukey's multiple comparisons test (*P* < 0.05).
**Figure S10.** Sequence analysis, phylogenetic analysis, subcellular localization and expression of apple *MdVQ22*. (A) Amino acid sequence alignment of apple MdVQ22 and its homologous genes in other species. The red box represents the FxxxVQxFTG domain. (B) Phylogenetic analysis of *MdVQ22* and its homologous genes. The accession numbers are displayed in parentheses. At, *Arabidopsis thaliana*; Me, *Manihot esculenta*; Pa, *Prunus armeniaca*; Md, *Malus domestica*; Pb, *Pyrus bretschneideri*; Pp, *Prunus persica*; Os, *Oryza sativa*; Ta, *Triticum aestivum*. The scale bar represents the replacement frequency of each amino acid site of the genome. (C) Subcellular localization of apple MdVQ22. Bars = 25 μm; DAPI, 4′,6‐diamidino‐2‐phenylindole; GFP, green fluorescent protein. (D) Tissue specific expression analysis of *MdVQ22* in apple rootstock R5‐5. The error bars represent the SD of three biological replicates (*n =* 3). Different letters indicate significant differences between different tissues, according to one‐way ANOVA and Tukey's test (*P* < 0.05). (E) Relative expression levels of *MdVQ22* in R5‐5 and M9T337 under salt treatment. The error bars represent the SD of three biological replicates (*n =* 3). Statistical significance was determined by two‐way ANOVA followed by Tukey's multiple comparisons test (*P* < 0.05).
**Figure S11.** TRV2‐*MdVQ22* transgenic apple plants maintain photosystem II activity under salt stress. (A) Chlorophyll fluorescence images, (B) F_0_ value, (C) Fm value, (D) Fv/Fm ratios, (E) Y(II), (F) Y(NPQ), and (G) Y(NO) of TRV2‐*VQ22* and TRV2 control apple plants under salt treatment. The error bars represent the SD of three biological replicates (*n =* 3). The experiments were repeated three times with similar results. Asterisks indicate significant differences (*P < 0.05, **P < 0.01) as determined by Student's t‐test.
**Figure S12.** The VQ22‐WRKY33 module acts as a conserved regulatory hub in the salt stress response of Populus. (A–D) Expression patterns of orthologous *PtVQ22* (A), *PtWRKY33* (B), *PtPER55* (C), and *PtDREB1* (D) in *Populus* 84 K under salt stress. (E) Amino acid sequence alignment of apple MdVQ22 and its ortholog PtVQ22 in *Populus* 84 K. The red box represents the FxxxVQxFTG domain. (F) Amino acid sequence alignment of apple MdWRKY33d and its ortholog PtWRKY33 in *Populus* 84 K. The red box represents the WRKYGQK domain. (G) Y2H assay to detect the interactions between PtWRKY33^(209–578)^ and PtVQ22 in yeast cells. pGBKT7‐53 served as positive control. Empty BD bait vector plus empty AD prey vector, empty BD bait vector plus AD‐PtVQ22 fusion prey vector and pGBKT7‐Lam served as negative controls. The experiments were repeated three times with similar results. A representative photograph is shown here. (H‐I) Relative expression of *PtDREB1* (H) and *PtPER55* (I) in *MdWRKY33d‐*overexpressing transgenic *Populus* lines (OE33d‐2 and OE33d‐6) and WT (84 K). The error bars represent the SD of three biological replicates (*n =* 3). Asterisks indicate significant differences (*P < 0.05, **P < 0.01) as determined by Student's t‐test.

**Table S1.** Primer sequences used in this study.

## Data Availability

The RNA‐seq raw sequence data reported in this paper have been deposited in the Genome Sequence Archive (GSA) in the National Genomics Data Center (NGDC), China National Center for Bioinformation (CNCB) / Beijing Institute of Genomics (BIG), Chinese Academy of Sciences (CAS), under accession number CRA044348 and are publicly accessible at https://ngdc.cncb.ac.cn/gsa/browse/CRA044348.
