# Supplementary material for: The MdVQ22‐MdWRKY33d complex regulates MdPER55 and MdDREB1D expression to modulate apple salt tolerance by reactive oxygen species scavenging
Source: Plant J. 2026 Oct 23;128(2):e71191. doi: 10.1111/tpj.71191 (PMC13691617; doi:10.1111/tpj.71191)
Supplement: Supplementary file 1 [file TPJ-128-0-s001.docx]

Figure S1. Phenotypic and physiological changes in two apple rootstock varieties under normal conditions and salt stress. (A) Representative phenotypic images of salt-sensitive rootstock M9T337 and salt-tolerant rootstock R5-5 at 15 d, 20 d, and 25 d under normal conditions and 150 mM NaCl. Scale bar=1 cm. (B)The net photosynthetic rate (Pn), (C) transpiration rate (E), (D) stomatal conductance (g_s_), and (E) intercellular carbon dioxide concentration (Ci) of both M9T337 and R5-5 under normal conditions and 150 mM NaCl at 25 d. (F) Contents of K^+^, (G)Na+, and (H) the Na^+^/K^+^ ratio (M) in the shoots of M9T337 and R5-5 under normal conditions and 150 mM NaCl at 25 d. Error bars represent SD of three biological replicates (*n* = 3). Significant differences were analyzed by ANOVA with Tukey's multiple range test (*P*< 0.05) and are indicated with different letters.

Figure S2. Phenotypic and physiological changes in the roots of two apple rootstock varieties under normal conditions and salt stress. (A) Representative phenotypic images of the roots of salt-sensitive rootstock M9T337 and salt-tolerant rootstock R5-5 at 25 d under normal conditions and 150 mM NaCl, respectively. Scale bar =1 cm. (B-E) Measurement of root volume (B), root surface area (C), root length (D) and root activity (E) of two apple rootstock varieties under normal conditions and salt stress. (F-J) REC (F), contents of MDA (G), K^+^ (H), and Na^+^ (I), and the Na^+^/K^+^ ratio (J) in the roots of M9T337 and R5-5 under normal conditions and 150 mM NaCl at 25 d. Error bars represent SD of three biological replicates (*n* = 3). Significant differences were analyzed by ANOVA with Tukey's multiple range test (P< 0.05) and are indicated with different letters.

Figure S3 Sequence analysis, phylogenetic analysis, tissue-specific expression, and subcellular localization of apple MdWRKY33d. (A) Phylogenetic analysis of MD12G1181000 and MD04G1167700 and their homologous genes in *Arabidopsis thaliana*. The scale bar represents the replacement frequency of each amino acid site in the genome. (B) Amino acid sequence alignment of apple MD12G1181000 and MD04G1167700 and their homologous genes in other species. The red box represents the WRKYGQK domain. At, *Arabidopsis thaliana*; Pb, *Pyrus bretschneideri*; Os, *Oryza sativa*; Vv, *Vitis vinifera*. (C) Subcellular localization of apple MdWRKY33d. Bars = 125 μm.(D) Tissue-specific expression analysis of *MdWRKY33d* in apple rootstock R5-5. Error bars represent SD of three biological replicates (*n* = 3). Different letters indicate significant differences between different tissues, according to one-way ANOVA and Tukey’s test (P < 0.05).

Figure S4 Relative expression levels of *MdWRKY33d* in 84K and transgenic lines. Error bars represent SD of three biological replicates (*n* = 3). Asterisks indicate significant differences (***P* < 0.01) as determined by Student's t-test.

Figure S5 Relative expression of *MdWRKY33d* and *MdVQ22* in WT and transgenic apple calli lines. (A,B) Relative expression of *MdWRKY33d* in MdWRKY33d-OE (A) and RNAi lines (B). (C) Relative expression of *MdVQ22* in MdVQ22-OE lines. (D) Relative expression of *MdWRKY33d* in MdWRKY33d/MdVQ22 co-overexpressing lines. Error bars represent SD of three biological replicates (*n* = 3). Asterisks indicate significant differences (**P* < 0.05, ***P* < 0.01) as determined by Student's t-test.

Figure S6 *MdWRKY33d* improves salt stress tolerance of transgenic apple calli by scavenging ROS. (A) Morphological traits of MdWRKY33d-OE (OE33d-3 and OE33d-7), MdWRKY33d-RNAi (RNAi33d-13 and RNAi33d-15), and WT before and after salt treatment (150 mM for 20d and 25d). (B) Fresh weight, (C) REC, (D) MDA, (E) Proline content, (F) H_2_O_2_ content, (G) O^2−^. content, (H) NBT and DAB staining, (I) SOD activity, (J) POD activity and (K) CAT activity of MdWRKY33d-OE (OE33d-3 and OE33d-7), MdWRKY33d-RNAi (RNAi33d-13 and RNAi33d-15), and WT after 150 mM salt treatment for 25d. Error bars represent SD from independent biological replicates (*n* = 6 for B; *n* = 3 for all other panels). Different letters indicate significant differences between different tissues, according to one-way ANOVA and Tukey’s test (P < 0.05).

Figure S7 (A)Differentially expressed analysis of *DREB* genes in the transcriptome of R5-5 (salt‐resistant) and T337 (salt‐sensitive) roots salt treatment. (B) Phylogenetic analysis of *MdDREB1D* and its homologous genes in *Arabidopsis thaliana*. The scale bar represents the replacement frequency of each amino acid site in the genome. (C) Relative expression levels of *MdDERB1D* in R5-5 (salt‐resistant) and T337 (salt‐sensitive) under salt treatment. The error bars represent the SD of three biological replicates (*n* = 3). Statistical significance was determined by two-way ANOVA followed by Tukey’s multiple comparisons test (*P* < 0.05).

Figure S8 The expression levels of the *MdDREB1D* gene in control plants and transgenic lines under both normal and salt-stress conditions. (A) *MdWRKY33d* overexpression transgenic apple calli and WT. (B) pCAMBIA2300-*WRKY33d* and pCAMBIA2300 transgenic apple seedlings. (C) TRV2-*WRKY33d* and TRV2 transgenic apple seedlings. The error bars represent the SD of three independent experiments (*n* = 3). Asterisks indicate significant differences (**P* < 0.05, ***P* < 0.01) as determined by Student's t-test.

Figure S9 *MdPER55* improves salt stress tolerance of transgenic apple calli by scavenging ROS. (A) Volcano plots of differentially expressed genes. differential genes for *MdWRKY33d* transgenic apple calli OE-7 and WT. (P < 0.05 and |log2(fold change)|> 2). (B) KEGG analysis showing differential gene enrichment terms for OE-7 and WT. (C) Differentially expressed analysis of *PER* genes in the transcriptome of *MdWRKY33d* transgenic apple calli OE-7 and WT (P < 0.05 and |log2(fold change)|> 2). (D) Relative expression of *MdPER55* in *MdWRKY33d* overexpression transgenic apple calli and WT. (E) Relative expression of *MdPER55* in pCAMBIA2300-WRKY33d and pCAMBIA2300 transgenic apple seedlings. (F) Relative expression of *MdPER55* in TRV2-WRKY33d and TRV2 transgenic apple seedlings. (G) Relative expression of *MdPER55* in *MdDREB1D* overexpression transgenic apple calli and WT. (H) Relative expression levels of *MdPER55* in R5-5 (salt‐resistant) and T337 (salt‐sensitive) under salt treatment. (I) *MdPER55* improves salt stress tolerance of apple calli. Scale bar=1 cm. (J) *MdPER55* expression levels, (K) REC, (L) O_2_^−^. content, (M) H_2_O_2_ content, (N) POD activity in *MdPER55*-OE and WT after 150 mM salt treatment for 25d. (O) Relative expression of *MdDREB1D* in *MdPER55* overexpression transgenic apple calli and WT. The error bars represent the SD of three independent biological experiments (*n* = 3). Asterisks indicate significant differences (**P* < 0.05, ***P* < 0.01) as determined by Student's t-test. For (H), statistical significance was determined by two-way ANOVA followed by Tukey’s multiple comparisons test (*P* < 0.05).

Figure S10. Sequence analysis, phylogenetic analysis, subcellular localization and expression of apple *MdVQ22*. (A) Amino acid sequence alignment of apple *MdVQ22* and its homologous genes in other species. The red box represents the FxxxVQxFTG domain. (B) Phylogenetic analysis of *MdVQ22* and its homologous genes. The accession numbers are displayed in parentheses. At, *Arabidopsis thaliana*; Me,*Manihot esculenta*; Pa, *Prunus armeniaca*; Md, *Malus domestica*; Pb, *Pyrus bretschneideri*; Pp, *Prunus persica*; Os, *Oryza sativa*; Ta, *Triticum aestivum*. The scale bar represents the replacement frequency of each amino acid site of the genome. (C) Subcellular localization of apple MdVQ22. Bars=25 μm; DAPI, 4′,6-diamidino-2-phenylindole; GFP, green fluorescent protein. (D) Tissue specific expression analysis of *MdVQ22* in apple rootstock R5-5. The error bars represent the SD of three biological replicates (*n =* 3). Different letters indicate significant differences between different tissues, according to one-way ANOVA and Tukey’s test (P < 0.05). (E) Relative expression levels of *MdVQ22* in R5-5 (salt‐resistant) and T337 (salt‐sensitive) under salt treatment. The error bars represent the SD of three biological replicates (*n =* 3). Statistical significance was determined by two-way ANOVA followed by Tukey’s multiple comparisons test (*P* < 0.05).

Figure S11. TRV2-*MdVQ22* transgenic apple plants maintain photosystem II activity under salt stress.(A) Chlorophyll fluorescence images, (B) F0 value, (C) Fm value, (D) Fv/Fm ratios, (E) Y(II), (F) Y(NPQ), and (G) Y(NO) of TRV2-VQ22 and TRV2 control apple plants under salt treatment. The error bars represent the SD of three biological replicates (*n =* 3). The experiments were repeated three times with similar results. Asterisks indicate significant differences (**P* < 0.05, ***P* < 0.01) as determined by Student's t-test.

Figure S12.The VQ22-WRKY33 module acts as a conserved regulatory hub in the salt stress response of Populus. (A-D) Expression patterns of orthologous *PtVQ22* (A), *PtWRKY33* (B), *PtPER55* (C), and *PtDREB1* (D) in *Populus* 84K under salt stress. (E) Amino acid sequence alignment of apple MdVQ22 and its ortholog PtVQ22 in *Populus* 84K. The red box represents the FxxxVQxFTG domain. (F) Amino acid sequence alignment of apple MdWRKY33d and its ortholog PtWRKY33 in *Populus* 84K. The red box represents the WRKYGQK domain. (G) Y2H assay to detect the interactions between PtWRKY33^(209-578)^ and PtVQ22 in yeast cells. pGBKT7-53 served as positive control. Empty BD bait vector plus empty AD prey vector, empty BD bait vector plus AD-PtVQ22 fusion prey vector and pGBKT7-Lam served as negative controls. The experiments were repeated three times with similar results. A representative photograph is shown here. (H-I) Relative expression of *PtDREB1* (H) and *PtPER55* (I) in *MdWRKY33d-*overexpressing transgenic *Populus* lines (OE33d-2 and OE33d-6) and WT (84K). The error bars represent the SD of three biological replicates (*n =* 3). Asterisks indicate significant differences (**P* < 0.05, ***P* < 0.01) as determined by Student's t-test.
